# *Erk1* and *Erk2* Regulate Endothelial Cell Proliferation and Migration during Mouse Embryonic Angiogenesis

**DOI:** 10.1371/journal.pone.0008283

**Published:** 2009-12-14

**Authors:** Ruchika Srinivasan, Tahera Zabuawala, Hong Huang, Jianying Zhang, Parul Gulati, Soledad Fernandez, J. Colleen Karlo, Gary E. Landreth, Gustavo Leone, Michael C. Ostrowski

**Affiliations:** 1 Department of Molecular and Cellular Biochemistry, The Ohio State University, Columbus, Ohio, United States of America; 2 Tumor Microenvironment Program, The Comprehensive Cancer Center, The Ohio State University, Columbus, Ohio, United States of America; 3 Center for Cardiovascular Medicine, Nationwide Children's Hospital, Columbus, Ohio, United States of America; 4 Center for Biostatistics, The Ohio State University, Columbus, Ohio, United States of America; 5 Department of Cellular and Molecular Neuroscience, Case Western Reserve University, Cleveland, Ohio, United States of America; 6 Department of Molecular Virology, Immunology and Medical Genetics, and Department of Molecular Genetics, The Ohio State University, Columbus, Ohio, United States of America; CNRS UMR6543 - Université de Nice - Sophia Antipolis, France

## Abstract

Angiogenesis is a complex process orchestrated by both growth factors and cell adhesion and is initiated by focal degradation of the vascular basement membrane with subsequent migration and proliferation of endothelial cells. The Ras/Raf/MEK/ERK pathway is required for EC function during angiogenesis. Although *in vitro* studies implicate ERK1 and ERK2 in endothelial cell survival, their precise role in angiogenesis *in vivo* remains poorly defined. *Cre/loxP* technology was used to inactivate *Erk1* and *Erk2* in endothelial cells during murine development, resulting in embryonic lethality due to severely reduced angiogenesis. Deletion of *Erk1* and *Erk2* in primary endothelial cells resulted in decreased cell proliferation and migration, but not in increased apoptosis. Expression of key cell cycle regulators was diminished in the double knockout cells, and decreased DNA synthesis could be observed in endothelial cells during embryogenesis. Interestingly, both Paxillin and Focal Adhesion Kinase were expressed at lower levels in endothelial cells lacking *Erk1* and *Erk2* both *in vivo* and *in vitro*, leading to defects in the organization of the cytoskeleton and in cell motility. The regulation of Paxillin and Focal Adhesion Kinase expression occurred post-transcriptionally. These results demonstrate that ERK1 and ERK2 coordinate endothelial cell proliferation and migration during angiogenesis.

## Introduction

Angiogenesis is a multi-step process involving a concerted remodeling of the primitive vascular plexus to a mature functional vascular network [1]. It is a fundamental process in normal growth and development. It is also an essential element in processes such as wound healing and inflammation, and in many pathological conditions, such as cancer. Soluble factors including vascular endothelial growth factor (VEGF) play key roles in this initial process, but cell adhesion/extracellular matrix interactions are also critical determinants of endothelial cell (EC) migration and proliferation [2].

Of the array of signaling events activated in response to VEGF and cell adhesion, the Ras/Raf/MEK/ERK1/2 pathway is known to play a prominent role in EC function [3], [4], [5], [6], [7]. Targeted deletion of genes in the Ras pathway, for example, *B-Raf*, *MEK1* and *Ras-GAP*, leads to vascular defects during embryogenesis [8], [9], [10], [11]. However, *Erk1^−/−^* mice are viable with no angiogenic phenotypes [12], [13] and *Erk2^−/−^* mice are embryonic lethal before vascularization and angiogenesis occur [14], [15], [16]. A large body of work demonstrates that the mitogen activated protein kinases ERK1 and ERK2 (ERK1/2) are critical for angiogenesis: however, the data is largely based on cell culture studies using dominant negative genes, siRNA and small molecule inhibitors to antagonize the kinases that lie upstream of ERK in the signaling pathway [17], [18], [19], [20], [21], [22]. These *in vitro* studies implicate the Ras/Raf pathway, and by extension ERK1/2, in EC survival and motility [17], [18], [19], [20], [21], [22]. However, besides Erk, many other signaling cascades such as src, FAK, PI3K/Akt, p38MAPK and JNK function downstream of growth factor and cell adhesion receptors, and many of these pathways have been implicated in the same biological processes [23], [24], [25], [26], [27], [28], [29], [30].

In order to better understand how ERK1 and ERK2 regulate angiogenesis, we used a genetic approach using *Cre/loxP* technology to conditionally delete *Erk2* in *Erk1*-null mice using the EC specific *Tie2-Cre* [31]. Embryos lacking *Erk1/2* in EC died *in utero* at E10.5 due to reduced angiogenesis both in the yolk sac and embryo proper. Gene expression profiling of isolated aortic EC identified cell cycle and cell migration as the principal biological processes affected in the double mutant EC. Consistent with the microarray profiling, EC lacking ERK1/2 show highly reduced proliferation and migration both *in vivo* and *in vitro*. The mechanism by which ERK1/2 regulated cell proliferation involved transcriptional regulation of genes necessary for cell cycle, including CyclinA, CyclinB, CyclinD1, CyclinE and c-Myc. Additionally ERK1/2 regulated the abundance of proteins like Paxillin and Focal Adhesion Kinase that are required for cell migration. These results reveal a redundant role for *Erk1* and *Erk2* in coordinating EC proliferation and migration, two processes necessary for embryonic angiogenesis.

## Materials and Methods

### Mice

*Erk1-null* and *Erk2^fl^* mice were described previously [12], [32]. The *Erk2^fl^* mice harbor *loxP* sites flanking exon 2. Cre-mediated recombination of this site results in the generation of the *Erk2-null* or *Erk2-KO* allele. *Tie2-Cre* mice were described previously [31]. All animals were maintained on pure C57/BL6 background (5 generations). Mice were genotyped by PCR. Primers and conditions used are available upon request. Use and care of mice in this study were approved by the Ohio State University Institutional Animal Care and Use Committee.

### Isolation of endothelial cells

Mouse aortic and lung EC were isolated from *Erk1^−/−^;Erk2fl^/fl^* and *Erk1^+/−^;Erk2^fl/f^* mice as previously described [33], [34]. Aortic and lung EC populations were labeled with 5 µg/mL Di-I-acetylated low-density lipoprotein (Di-I-Ac-LDL, Upstate) and enriched by fluorescent activated cell sorting as the final step of purification. The purified EC populations were characterized and maintained in complete EC medium (Dulbecco modifies Eagle medium (DMEM)-F12 with 20% heat inactivated FBS plus penicillin-streptomycin, 30 µg/ml endothelial cell growth supplement (Upstate Biotechnology) and 10 U/ml heparin (Sigma-Aldrich)) in 37°C incubator at 5% CO_2_.

### Lentiviral Transduction

Duplicate cultures of *Erk1^−/−^;Erk2fl^/fl^* and *Erk1^+/−^;Erk2^fl/f^* EC (5×10^5^ cells) were cultured overnight and infected with ecotropic lentivirus with and without PGK-Cre, to generate control and double mutant (DKO) EC, respectively (pHAGE-IRES-GFP vectors used were a gift from Dr.N.Danial's laboratory at Harvard University). Infections of the duplicate cultures were performed as described previously [35]. The infected cells were harvested 72 hrs post-infection, when the endogenous pool of ERK2 present before infection was sufficiently depleted and morphological differences between control and DKO cells are first noted. All subsequent experiments were performed on the duplicate cultures, one infected with Cre and one without, beginning at 72 hrs post-infection.

### Quantitative real-time PCR

Total RNA was extracted from aortic and lung EC 72 hrs post viral infection by TRIzol (Invitrogen) according to the manufacturers' instructions. Samples were analyzed by q-PCR as previously described [35]. Students t-test was used to determine the statistical significance of the expression differences between the DKO and control genotypes.

### Microarray Analysis

A detailed description of microarray analysis is presented in Table S3. Briefly, cells used for this study were generated from three independent pairs of aortic EC infected with lentiviral-*eGFP* with and without *Cre*. RNA was extracted from control and DKO EC at 72 hrs post-lentivirus infection. Microarray was performed with these RNA samples on Affymetrix Mouse Exon v1.0 ST Array GPL6193 platform. RMA method was applied to the primary data to correct the technical bias and summarize gene expression values over probe-sets [36]. The gene expression differences were then compared between EC with or without ERK1/2. The gene list was filtered based on the fold change in expression between the two genotypes. Targets showing >4-fold significant (p<0.05) change in expression in the DKO compared to control EC were subjected to DAVID Annotation Analysis (http://david.abcc.ncifcrf.gov/).

### Histology and Immunostaining

Whole-mount CD31 staining on embryos was performed as previously described [37]. Embryos were fixed in 4% paraformaldehyde (PFA) overnight, paraffin or OCT embedded and 5 µm sections were prepared. Immunostaining was performed as previously described [35], [38]. Primary antibodies used were rat anti- CD31 (MEC13.3, BD Phamingen), rabbit anti-ERK1/2 (Santa Cruz), rabbit anti-cleaved Caspase-3 (Cell Signaling), mouse anti-BrDU (DAKO Cytomation), rabbit anti-Paxillin (Abcam), rabbit anti-FAK (Cell Signaling) and rabbit anti-pY397-FAK (Abcam). Biotinylated secondary antibodies used were from BD Biosciences. The antibody staining was imaged using an Axioscope 40 microscope (Zeiss) equipped with an Axiocam HRc camera (Zeiss). Immunostaining was quantified in embryonic blood vessels in the head, lining the heart, the dorsal aorta, the cardinal vein, and the intersomatic vessels. At least 10 vessels (∼400 EC) were counted per embryo in each group and were quantified using Metamorph 6.0. For non-EC, Metamorph 6.0 was used to count and quantify staining in all embryonic cell types (such as smooth muscle cells, epithelial cells, cardiomyocytes), especially cells adjacent to blood vessels. Approximately 1500 non-EC cells per embryo in each genetic group were counted and scored.

EC plated on Fibronectin (FN)-coated dishes (10 µg/ml) were used for double immunofluorescence assays. Alexa-594 conjugated donkey anti-mouse (Invitrogen), Alexa-488/594 conjugated secondary antibodies (Invitrogen) were used for immunofluorescence analysis. Images of stained EC were acquired using a Zeiss 510 META laser scanning confocal microscope. For the Erk1*^−^* ^/*−*^;Erk2*^−^* ^/*−*^ DKO cells, which express low levels of GFP through an IRES sequence, the FITC intensity of unstained DKO ECs was set as background. Using this as the basal level, FITC expression in Paxillin immunostained DKO EC was evaluated. Results presented have the GFP background signal filtered. To confirm the results, in separate single label experiments Paxillin was stained using the Alexa598 red conjugate (data not shown). Phalloidin labeling was performed on EC according to the manufacturers' instructions (Invitrogen). Immunostaining of EC in culture was performed 72 hrs post viral infection.

### Western Blotting

Total protein was extracted from at least two control and DKO EC 72 hours post viral infection and western blot was performed as previously described [35]. Primary antibodies raised in rabbit against ERK1/2, CyclinD1, CyclinA, CyclinE, CyclinB1, CDK4 and CDK1, and mouse anti-c-Myc were purchased from Santa Cruz Biotechnology. Other primary antibodies used were rabbit anti-Paxillin (Abcam), rabbit pS473-AKT and rabbit anti-FAK (Cell Signaling) and rabbit anti-pY397-FAK (Abcam).

### Cell Proliferation and Migration assays

72 hrs post viral infection, 3×10^5^ EC were plated overnight with fresh media and subsequently incubated with 3 µg/ml BrDU (Sigma) for 2 hrs in 37°C incubator at 5%CO_2_. The cells were then fixed and BrDU staining was performed as previously described [38]. For all migration assays, EC obtained 72 hrs post viral infection were used. For Scratch Wound Assay, EC were cultured an additional 24 hrs until confluent, incubated with 15 ng/ml MitomycinC (Sigma) and wounded across the well surface with a 10-µl standard pipette tip. The wounded monolayers were then imaged over 24 hrs under the Live Imaging Zeiss microscope. Cell migration was calculated as the number of EC migrating into the wound area at various time points indicated. Single cell migration track assays were performed as previously described and quantified using Metamorph 6.0 [39]. The vessel-forming ability of EC was characterized *in vitro* using a Matrigel assay. Briefly, 4×10^4^ EC were sandwiched between two layers of Matrigel (BD Biosciences, 10 mg/ml) and cultured in complete EC media. Tube formation was monitored over a period of a week.

### EC apoptosis and senescence assays

Serum starvation (0.1% serum for 24 hrs) was used to induce apoptosis in EC infected with lentivirus with and without Cre (72 hrs post infection). Apoptosis was assayed using fluorescence labeled LIVE/DEAD Cell Viability Kit (Invitrogen) according to the manufacturer's protocol and quantified using Metamorph6.0 as previous described [35]. Senescence assays were performed as previously described [40].

### Statistical Analysis

General Linear Models were used to analyze staining differences in immunostaining experiments. MINITAB was used for the analyses.

## Results

### Ablation of Erk1 and Erk2 in EC results in embryonic lethality and defective angiogenesis

In order to study the potential overlapping roles of *Erk1/2* in the development of the vascular system, a genetic approach employing *Cre/LoxP* technology was used to generate mice with both genes absent in EC. For this purpose we generated mice homozygous for a conditional *Erk2* allele, *Erk2^fl^* (with exon 2 flanked by *loxP* sites) [32], homozygous for the conventional knockout allele of *Erk1* [12], and containing the well-characterized *Tie2-Cre* transgene to effect recombination of *Erk2^fl^* in EC [31]. Mice with genotype *Erk1^−/−^;Erk2^fl/fl^;Tie2-Cre* (EC-DKO mice) were expected to be born at a frequency of 12.5% from the initial breeding scheme employed (see Figure S1A for breeding schemes). However, no mutant EC-DKO mice were born, suggesting embryonic lethality; other expected genotypes were found at the predicted ratios, indicating that one copy of either *Erk1* or *Erk2* in EC was sufficient for embryonic development (Table S1).

**Figure 1:**
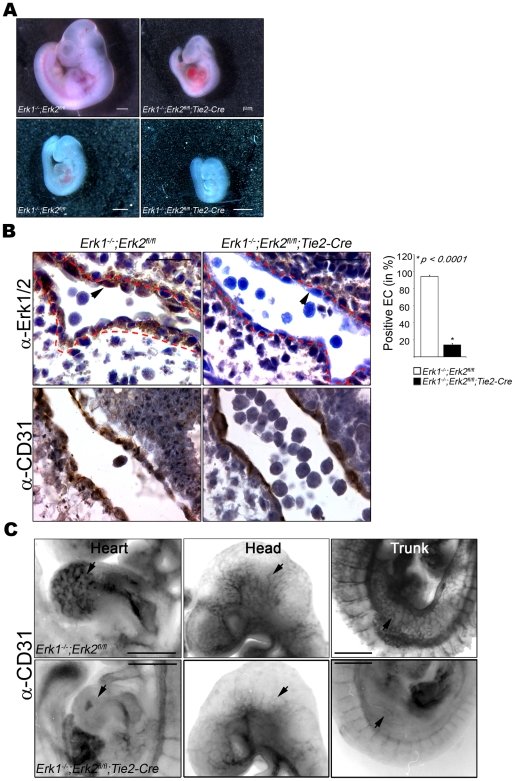
Embryonic Lethality and Defective Angiogenesis in *Erk1* *^−^* ^*/−*^ *;Erk2^fl/fl^;Tie2-Cre* mutant embryos. (A) E10.5 day (top) and E9.5 (bottom) embryos; control embryo is at the left, DKO at the right. Bars = 0.5 mm. (B) Consecutive paraffin sections of control (left) and *Erk1^−/−^;Erk2^fl/fl^;Tie2-Cre* (EC-DKO) (right) embryos were stained with anti-ERK1/2 (top) and anti-CD31 (bottom) antibodies. Representative data for E9.5 embryo are shown; a total of 4 embryos were analyzed. Red dotted lines mark the outer lining of the EC layer and arrowheads point to representative EC. Bars = 20 µm. The graphic panel indicates the ratio of ERK positive EC to total CD31-positive cells, expressed as percent positive EC. (C) E9.5 embryos analyzed by whole mount staining with anti-CD31 antibody. Controls are in the top row and EC-DKO mutants in the bottom row. Arrowheads highlight examples of blood vessel staining and branching in the controls that are reduced in the EC-DKO embryos. Bars = 0.5 mm.

To determine when lethality occurred, embryos obtained from timed matings were studied, demonstrating that EC-DKO embryos were alive at E9.5, when the *Tie2-Cre* transgene utilized in these studies becomes active in the embryo body [31], but no longer viable at E10.5 (Figure 1A; Table S1 and S2). The viable EC-DKO E9.5 embryos were smaller than the control littermates (Figure 1A). There was no significant difference in the number of somites present in the mutant mice compared to the controls (Figure S1B). Blood vessels were not visible in the yolk sac of the EC-DKO embryos at E9.5, consistent with the timing of expression of *Tie2-Cre* in yolk sac beginning at E7.5-8.5 [31] (Figure S1D). Furthermore, EC-DKO mutant hearts were much smaller with highly reduced cellularity and the endocardial layer was detached from myocardial trabeculae suggesting defective heart development and function (Figure S1E).

In order to verify that both *Erk1/2* were selectively deleted in EC, immunohistochemistry was performed on sections from viable E9.5 embryos using a pan-ERK1/2 antibody. While robust ERK1/2 expression was detected in EC present in controls, a selective 8-fold reduction of ERK1/2 expression was observed in EC in the EC-DKO mutants (Figure 1B). Notably, there was no significant difference in ERK1/2 expression in other embryonic non-EC types such as smooth muscle cells, epithelial cells, cardiomyocytes etc. (Figure S1C).

Whole mount CD31 staining was employed to determine the extent of angiogenic remodeling in the EC-DKO embryos, an analysis that revealed a striking reduction in vascular complexity in the EC-DKO embryos examined (n = 7) compared to controls (n = 10) at this stage (Figure 1C and Figure S1F). While the control littermates underwent extensive blood vessel branching and maturation at E9.5, the EC-DKO embryos had only large unbranched vessels, especially evident in the heart, head and trunk regions. Additionally, whole mount CD31 staining of EC-DKO yolk sacs (n = 4) revealed large vessels and decreased vascular density compared to the control littermates (n = 4), confirming that angiogenesis was impaired in this embryonic tissue (Figure S1D).

### Gene expression profiling of ERK1/2 deficient EC identified proliferation and migration as affected biological processes

**Figure 2:**
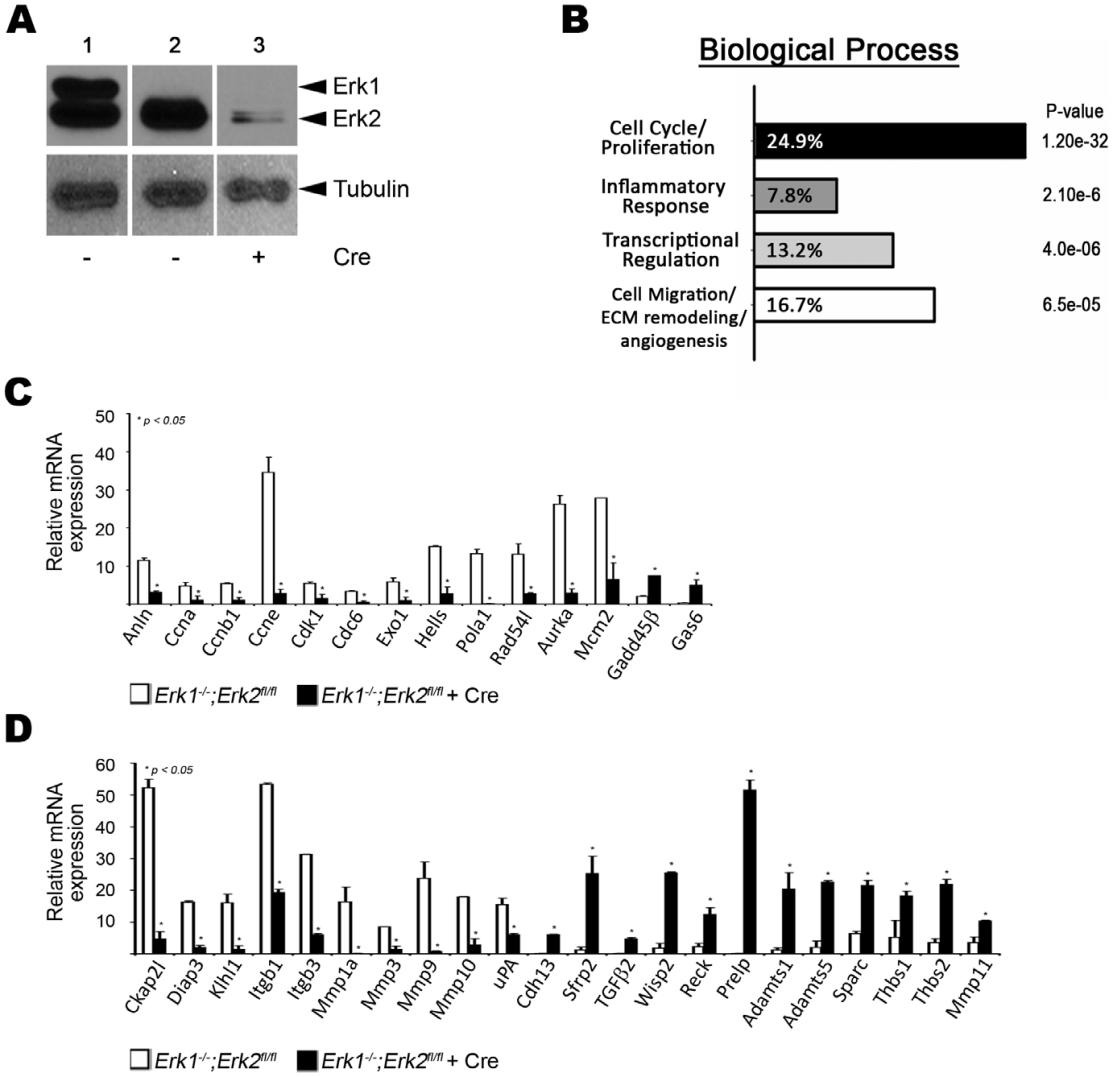
Loss of ERK1/2 in EC affects Cell cycle/proliferation and Cell migration/angiogenesis. (A) Protein expression of ERK1/2 in lentivirus infected aortic control and DKO EC by Western analysis. *Erk1^+/−^;Erk2^fl/fl^* (lane 1) and *Erk1^−/−^;Erk2^flfl^* without (lane 2) and with (lane 3) Cre. (B) Principal biological processes affected by the loss of ERK1/2 in aortic EC. Indicated is the % genes differentially expressed in DKO EC. (C and D) Gene expression analysis of cell cycle/proliferation (C) and cell migration/ECM remodeling/angiogenesis (D) regulators by q-PCR in cultured aortic EC with and without ERK1/2, n = 3.

To directly address the mechanisms that might be contributing to the lethality in the double mutant embryos, primary aortic EC and microvascular lung EC were isolated and cultured from *Erk1^−/−^;Erk2^fl/fl^* mice. The EC identity and relative purity of the cultured cells was confirmed by the uptake of fluorescent-labeled low density lipoprotein and CD31 staining (Figure S2A and S2B). The purified cell populations were infected with lentivirus expressing both *Cre* and *eGfp* to generate aortic EC lacking *Erk1* and *Erk2* (DKO EC). The efficiency of lentiviral-*Cre* mediated recombination was analyzed by Western analysis and indirect immunofluorescence, demonstrating a 10-fold reduction in ERK2 protein levels (Figure 2A and Figure S2C, respectively). Similar results were obtained with the lung microvascular cells (Figure S2D). Aortic and lung EC of genotype *Erk1^−/−^;Erk2^fl/fl^* infected with lentivirus without *Cre* were used as controls for subsequent experiments.

Subsequently, total RNA was isolated from DKO and control EC and subjected to gene expression profiling using the Affymetrix platform. The expression profiling was performed on three independent pairs of aortic EC cultures. RNA was isolated from DKO EC 72 hrs post lentiviral expression, sufficient time for endogenous ERK proteins to turn over. Analysis of the microarray data yielded 281 genes that showed 4-fold or greater, statistically significant changes in gene expression (Table S4). Of these, 144 genes were downregulated and 137 genes were upregulated. Gene ontology identified four principal biological processes affected by the loss of *Erk1/2* in EC: cell cycle/proliferation, cell migration/angiogenesis, transcriptional regulation and inflammatory response (Figure 2B).

To verify the microarray results, quantitative real-time RT-PCR (q-PCR) was performed on RNA prepared from an independently derived set of aortic EC. 35 genes with greater than four-fold differential expression that represented the major biological processes affected were selected for verification. In addition, we selected for q-PCR verification another 13 genes described in the literature to be regulated by ERK signaling (e.g. MMP9 and uPA), that were differentially expressed greater than two-fold in the microarray analysis (Table S5). All 48 genes tested by q-PCR were found to be significantly differentially expressed in DKO versus control aortic EC (Figure 2C-D; Figure S2E). EC-specific genes (*Cd31* and *Flt1*), EC-growth factor (*Vegf-c*), cell cycle regulators (*p27* and *p53*), and pro-apoptotic genes (*Caspase3*) were not significantly affected in the DKO EC (Figure S2F). Expression of many of the genes differentially regulated in DKO aortic EC was studied in the microvascular lung EC, revealing differential expression in this distinct type of EC as well (Figure S2G).

### Erk1 and Erk2 regulate EC proliferation *in vivo* and *in vitro*

**Figure 3:**
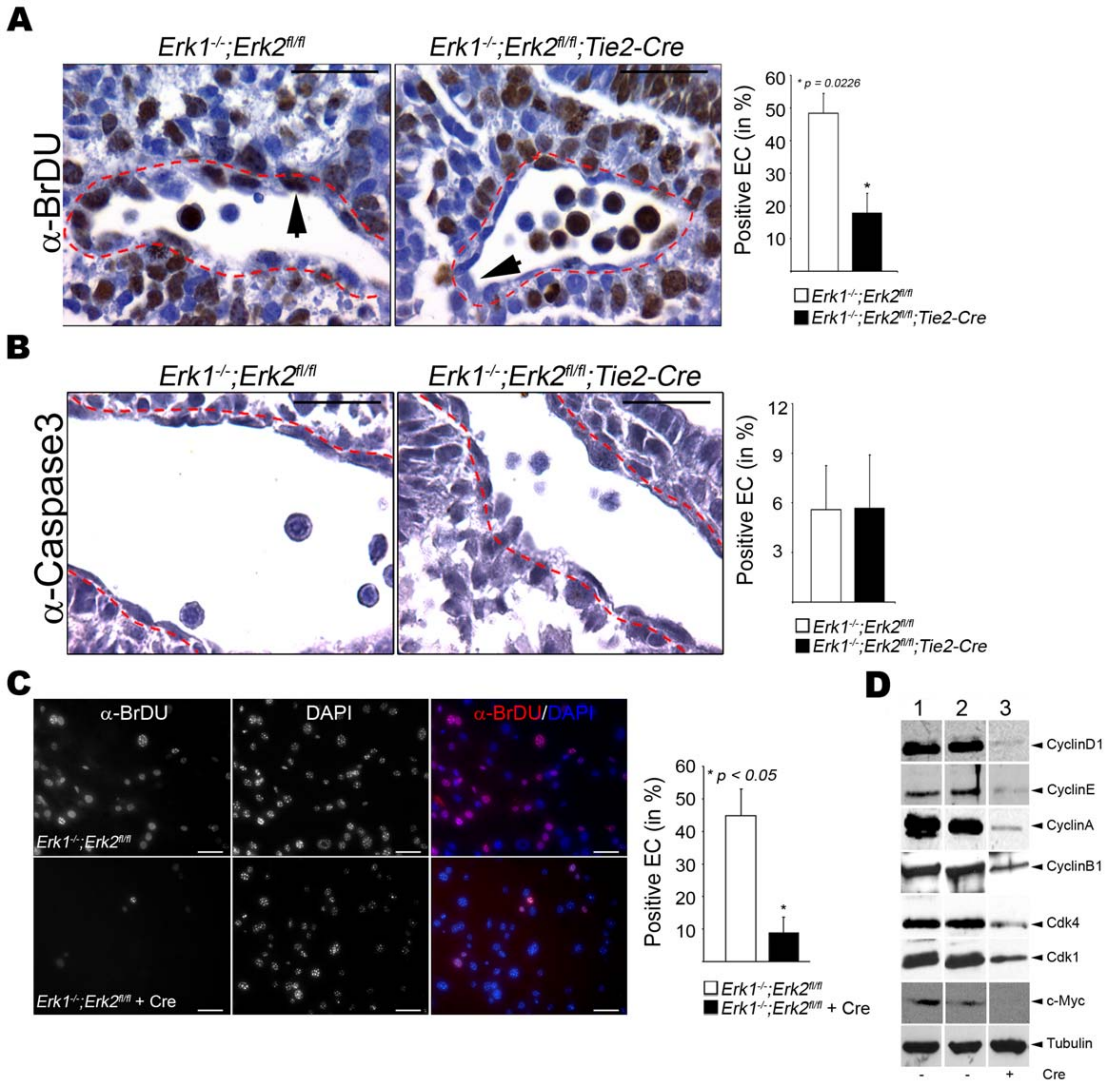
Reduced cell proliferation in ERK1/2 deficient EC. (A) Representative paraffin sections from E9.5 control (left, n = 4) and EC-DKO (right, n = 4) embryo sections stained for anti-BrDU. The red dotted lines indicate the outer lining of the EC layer and the arrowheads point to representative EC. The bar graph represents the fraction of BrDU positive to total EC. Bars = 20 µm. (B) Anti-cleaved Caspase-3 staining of representative paraffin sections from E9.5 control (left, n = 4) and EC-DKO (right, n = 4) embryo. The red dotted lines indicate the outer lining of the EC layer. The graphic panel indicates the fraction of EC staining for cleaved Caspase-3 to total EC. Bars = 20 µm. (C) Anti-BrDU staining on cultured aortic EC of the indicated genotype, without (top panel) and with (bottom panel) Cre. Red-BrDU and Blue-DaPI. The graph indicates the ratio of BrDU positive to total EC with 3 independent experiments represented. Bars = 20 µm. (D) Western blot analysis on lysates from *Erk1^+/−^;Erk2^fl/fl^*-Cre (lane 1), *Erk1^−/−^;Erk2^fl/fl^*-Cre (lane 2) and *Erk1^−/−^;Erk2^fl/fl^*+Cre for the antibodies indicated.

Since the expression profiling results predicted differences in cell proliferation and migration, these processes were selected for further analysis. EC proliferation in EC-DKO embryos was analyzed by employing a BrDU incorporation assay (Figure 3A). EC-DKO mutant embryos showed a selective 3-fold decrease in BrDU incorporation in EC compared to controls (Figure 3A). Importantly, there was no significant difference in BrDU incorporation in other non-EC types such as smooth muscle cells, epithelial cells, cardiomyocytes (Figure S3A). Because previous studies had implicated ERK1/2 in EC survival during angiogenesis, cleaved Caspase-3 immunostaining was used to determine if EC apoptosis was affected in EC-DKO embryos. The results demonstrated that apoptosis was low in EC in E9.5 embryos, with no significant difference between EC-DKO and control embryos (Figure 3B).

Consistent with the *in vivo* data, there was a 4.5-fold reduction in BrDU incorporation in DKO compared to control aortic EC *in vitro* (Figure 3C). DKO lung EC also showed ∼4-fold decrease in BrDU incorporation compared to wild-type controls (Figure S3B). Further, aortic EC with only one *Erk* allele do not show significant difference in BrDU incorporation compared to the controls (Figure S3C). This suggested that one copy of either *Erk* gene was sufficient to maintain normal EC proliferation. Additionally, consistent with *in vivo* results, there was no difference in apoptosis in DKO versus control aortic EC when stressed by serum deprivation (Figure S3D).

Western blotting was used to analyze the expression of several cell cycle regulators in aortic EC. A marked diminution of proteins regulating G1 to S phase progression was observed, including CyclinD1, CyclinE, CDK4 and c-Myc in the DKO aortic EC (Figure 3D). Interestingly, the expression of G2-M regulators such as CyclinA, CyclinB1 and CDK1 were also lower in DKO aortic EC (Figure 3D).

Deletion of *Erk1/2* in either aortic or lung EC *in vitro* led to marked morphological changes, including larger and flattened cells. To test whether cell senescence was being triggered by deletion of the *Erk* genes, we stained cells for SA-βgal activity, a biomarker of cellular senescence (Figure S3E). Neither DKO nor control aortic EC expressed SA-βgal under normal culture conditions; however, upon the induction of senescence by UV-irradiation, both DKO and control aortic EC stained for SA-βgal (Figure S3E). Thus, loss of ERK1/2 did not directly trigger cell senescence in cultured EC.

### *Erk1/2* ablation in EC results in reduced EC migration and tube formation *in vitro*

**Figure 4:**
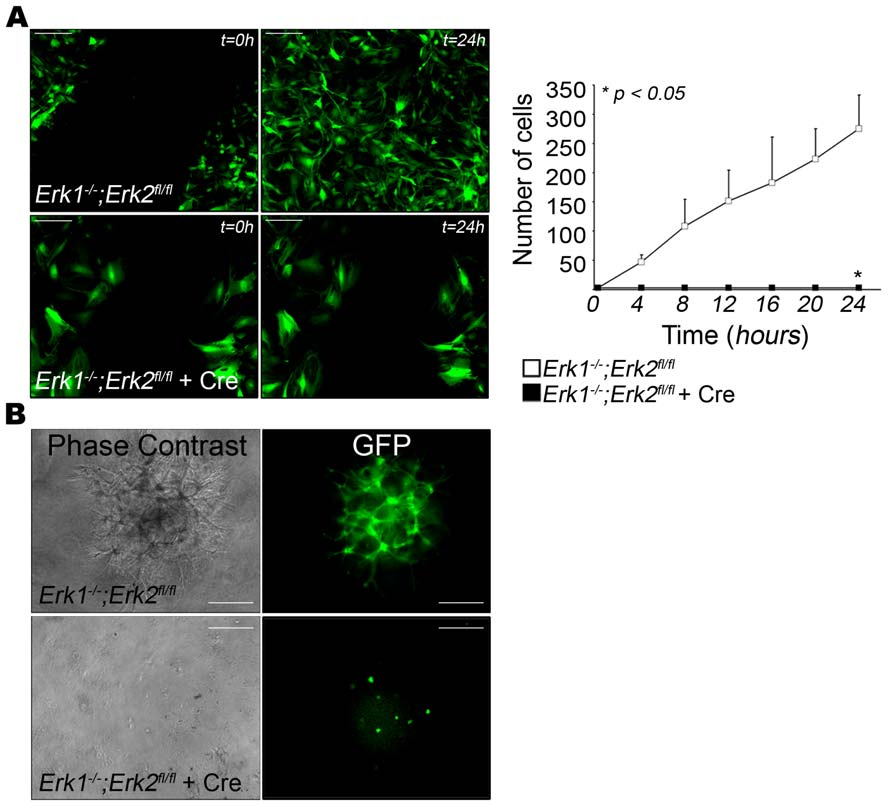
EC lacking ERK1/2 display reduced migration and invasion potential *in vitro*. (A) Confluent monolayer of control (top panel) and DKO (bottom panel) aortic EC were wounded and wound closure was monitored over 24 hrs. Representative results from t = 0 h (left panel) and t = 24 h (right panel) are shown (n = 3). The graph indicates the quantification of the number of cells migrating into the wound over the indicated time points. Bars = 40 µm. (B) *In vitro* matrigel sandwich assay showing invasion and tube-like formation by control aortic EC (top panel) that is absent in DKO EC (bottom panel). Left panel shows the phase contrast images and the right panel GFP images. Bars = 80 µm.

Three different assays that measure cell motility and invasiveness were used to examine the effect of *Erk1*/*2* deletion on cell migration. Initially, a monolayer scratch wound assay was performed. After wounding (and in the presence of MitomycinC), control EC efficiently migrated into the wounded area within 24 hrs (Figure 4A). In contrast, the DKO aortic EC failed to migrate into the wounded area (Figure 4A, Movies S1 and S2). Similar results were also observed with DKO lung EC (Figure S4A). Further, aortic EC with only one *Erk* allele did not show significant differences in motility in this assay (Figure S4B). As above, this result suggested that one copy of either *Erk* gene was sufficient for normal EC migration. The results of the scratch assays from DKO and control EC were confirmed using a single cell migration track assay in which migrating cells engulf fluorescent beads leaving a non-fluorescent track (Figure S4C). A 6-fold decrease in motility of the DKO aortic EC compared to control was measured in this assay. Cell invasiveness of DKO EC was examined in a Matrigel “sandwich” assay with EC placed between two layers of Matrigel. The DKO aortic EC failed to invade into the Matrigel and to form tube-like structures as observed for controls (Figure 4B).

### Erk1/2 deletion altered expression and localization of Paxillin and FAK

**Figure 5:**
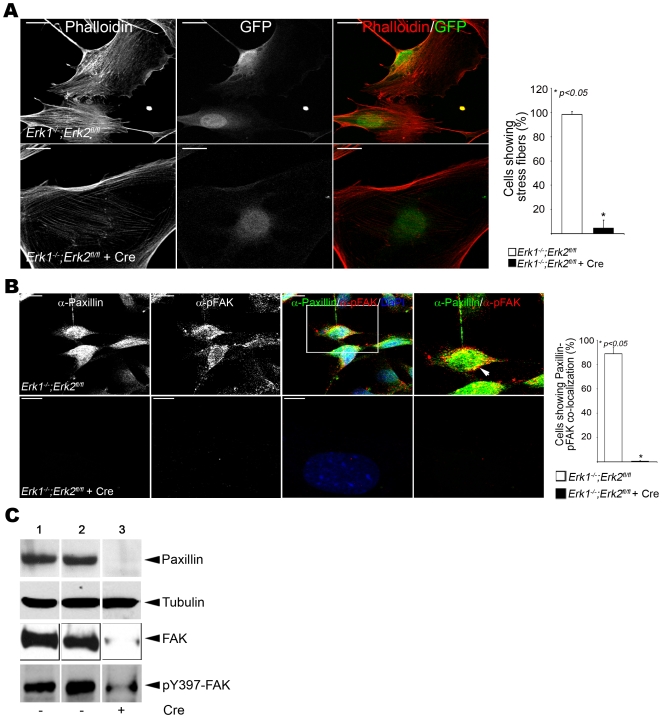
ERK1/2 deficient EC show altered actin organization and reduced expression of Paxillin and FAK *in vitro*. (A) Representative micrographs of phalloidin stained control (top) and DKO (bottom) aortic EC (n = 3). Red = Actin and Green = lentivirus infected cells. The bar graph represents % EC showing the presence of intra-cellular stress fibers. Bars = 20 µm. (B) Representative micrographs of Paxillin-pFAK membrane co-localization on FN-coated dishes in *Erk1^−/−^;Erk2^fl/fl^* (top) and *Erk1^−/−^;Erk2^fl/fl^*+Cre (bottom) aortic EC (n = 3). Red-Paxillin and Green-pY397 FAK. Boxed areas are enlarged to show membrane staining in EC. Graphic panel represents % EC showing Paxillin-pY397 FAK co-localization. Bars = 20 µm. (C) Western blot with indicated antibodies of lentivirus infected aortic EC. *Erk1^+/−^;Erk2^fl/fl^* (lane 1) and *Erk1^−/−^;Erk2^fl/fl^* without (lane 2) and with (lane 3) Cre.

Previous studies in EC have shown that ERK1/2 regulate the organization of the actin cytoskeleton and hence cell motility [19], [41], [42], [43]. Consistent with these studies, phalloidin stained DKO compared to control aortic EC demonstrated a dramatic loss of actin cytoskeletal organization, in particular the loss of intracellular stress fibers and marked peripheral accumulation of actin in both aortic and lung DKO EC compared to controls (Figure 5A and Figure S5A).

Paxillin and Focal Adhesion Kinase (FAK) are two factors indispensable for actin filament assembly, cell spreading and cell migration [44]. Indirect immunofluorescence was used to assess whether Paxillin and FAK localization were affected in DKO EC plated on fibronectin (FN) coated dishes. While the control EC demonstrated expression and co-localization of Paxillin and pY397-FAK at the membrane and within focal adhesions, staining was greatly diminished in DKO aortic EC and focal adhesions were not detected with these markers (Figure 5B). Consistent with the double immunofluorescence analysis, the expression of Paxillin and FAK (both total and pY397-FAK), were significantly reduced in DKO compared to control aortic EC as demonstrated by Western blotting (Figure 5C). The mRNA levels for both *Paxillin* and *Fak*, were unchanged in DKO EC compared to controls indicating that the changes in protein expression were due to post-transcriptional events (Figure S5B). The Paxillin/FAK complex activates the PI-3 Kinase pathway and AKT [45]. However, no difference in activated pS473-AKT was observed in the DKO aortic EC (Figure S5C).

**Figure 6:**
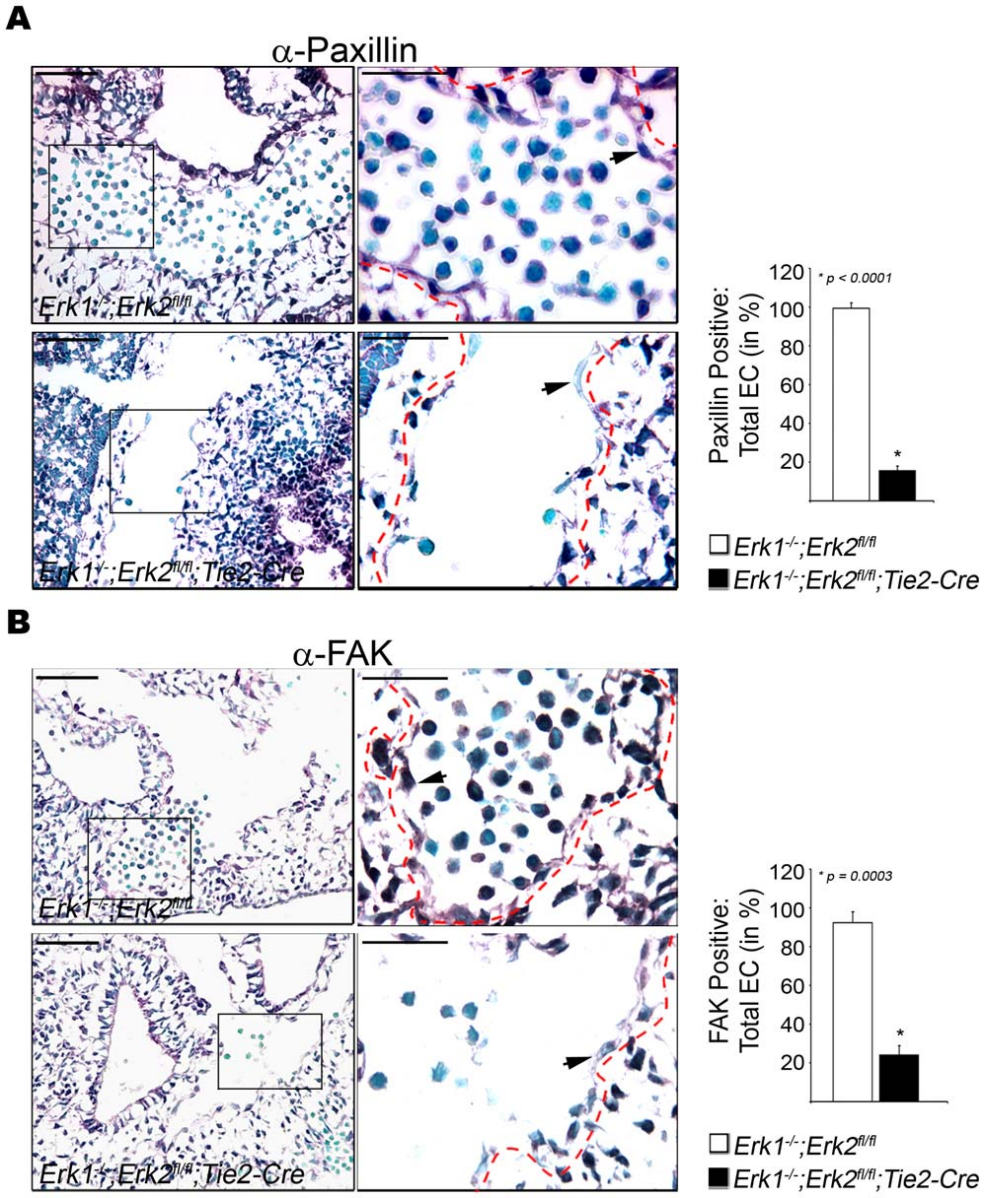
ERK1/2 deficient EC show reduced expression of Paxillin and FAK *in vivo*. (A) Representative cryosections of E9.5 *Erk1^−/−^;Erk2^fl/fl^* control (left) and *Erk1^−/−^;Erk2^fl/fl^ ;Tie2-Cre* (EC-DKO, right) embryos (n = 4 of each genotype) stained with anti-Paxillin. Graphic panel indicates the ratio of Paxillin positive area to total EC area in %. (B) Anti-FAK staining of representative cryosections from E9.5 *Erk1^−/−^;Erk2^fl/fl^* control (left, n = 4) and EC-DKO mutant (right, n = 4) embryo. Graphic panel indicates the ratio of FAK positive area to total EC area in %. Boxed areas are enlarged to show membrane staining in EC. The red dotted lines indicate the outer lining of the EC layer and the arrowheads point to representative EC. Lower magnification: Bars = 25 µm and Higher magnification: Bars = 20 µm.

To determine whether Paxillin and FAK were affected in EC-DKO embryos, immunohistochemistry was used to analyze their expression in E9.5 embryos. Importantly, EC-DKO embryos showed an 8-fold reduction in EC-specific staining for Paxillin and a 4-fold decrease in FAK staining (Figure 6A and 6B, respectively). There was no significant difference in Paxillin and FAK staining in the other embryonic non-EC types such as smooth muscle cells, epithelial cells, cardiomyocytes etc. (Figure S5D).

## Discussion

The combination of *in vivo* and *in vitro* approaches presented here reveal an EC autonomous, redundant role for ERK1/2 in embryonic angiogenesis. The combined results demonstrated that *Erk1/2* regulate both proliferation and migration of EC, two biological processes that are critical for angiogenesis. Surprisingly, cell apoptosis was not affected by ablation of *Erk1*/*2* in EC as predicted by previous *in vitro* studies. A plausible explanation for this discrepancy might be that in the previous *in vitro* studies upstream components of the RAS/ERK pathway were targeted, in particular RAF and MEK [2], [17], [20]. Thus, the actual role of ERK1/2 in apoptosis was inferred but not directly tested in these studies. This raises the possibility that targets of RAF and MEK other than ERK1/2 may be involved in the observed affects of the pathway on EC survival.

EC-specific deletion of *Erk2* in the *Erk1^−/−^* background resulted in embryonic lethality at E9.5-E10.5 and the viable EC-DKO E9.5 embryos were smaller than the control littermates. We speculate that the smaller size of EC-DKO embryos is due to *Tie2-Cre* mediated deletion of *Erk2* starting at E7.5 in the yolk sac EC, and at E8.5 in the embryonic aorta and common atrial chamber [31]. In addition, reduced blood vessel development in the embryo proper likely contributes to the smaller size and in combination with defective heart development results in lethality of the EC-DKO mutant embryos.

One mechanism underlying ERK1/2 function in EC involves regulation of the expression of FAK and Paxillin. ERK1/2 have been most frequently placed downstream of Src-FAK signaling in several studies [46], [47], but our results demonstrate that ERK1/2 can also act upstream to regulate FAK and Paxillin expression. One hypothesis to account for this result is that ERK activation by tyrosine kinases is required for efficient expression of FAK and Paxillin, which in turn leads to amplification of ERK signaling when integrin/adhesion pathways are engaged. An alternate mechanism by which ERK can function upstream of these signaling proteins is that, as demonstrated in hepatocytes, ERK phosphorylation of Paxillin at Ser83 enhances formation of Paxillin/FAK complexes in focal adhesions [48], [49]. However, the drastic reduction in FAK and Paxillin expression in EC lacking ERK1/2 don't allow the potential role of this mechanism in EC to be tested. The precise mechanism by which ERK regulates FAK and Paxillin remains to be determined, but could involve selective regulation of translation of these proteins, including potentially through the regulation of specific microRNAs, or by affected turnover rates for these proteins.

FAK signaling has been linked to EC proliferation as well as migration, suggesting that the loss of FAK expression could account for the major phenotypes observed when ERK1/2 are ablated in EC [45], [50]. However, in two separate studies *Tie2-Cre* mediated conditional knockout of *Fak* resulted in a less severe phenotype than reported here, with embryonic lethality occurring at E11.5 or later, and without angiogenic phenotypes at E9.5 when defects are obvious with *Erk1*/2 deletion [38], [51]. In addition, EC apoptosis was identified as a major phenotype in both of these previous studies. Thus, other targets of ERK1/2 in addition to FAK and Paxillin likely account for the overall phenotype observed.

The microarray analysis suggests that the nuclear function of ERK1/2 is also critical for their action in EC. For example, a number of genes involved in cell cycle regulation, in particular Cyclins, CDKs and c-Myc are among ERK targets that can account for cell proliferation defects in the double mutant mice. Similarly, the expression of several extracellular proteases (e.g., MMPs) involved in promoting cell motility and angiogenesis were downregulated while extracellular matrix components that inhibit angiogenesis (e.g., Thrombospondins 1 and 2) were increased by the absence of ERK1/2. Therefore, the ability of ERK1/2 to activate an assortment of transcription factors, either directly by phosphorylation or indirectly through activation of their expression, is key to their function in EC.

In summary, these *in vitro* and *in vivo* results demonstrate a redundant role for *Erk1* and *Erk2* in EC, and indicate that targets at both the cell membrane and in the nucleus account for the ability of these kinases to regulate EC functions that are necessary for embryonic angiogenesis.

## Supporting Information

Table S1 EMBRYONIC LETHALITY IN Erk1^−/−^; Erk2^fl/fl^; Tie2Cre DOUBLE MUTANT MICE
(0.05 MB DOC)

Table S2 EMBRYONIC LETHALITY IN *Erk1^−/−^; Erk2^fl/fl^; Tie2Cre* DOUBLE MUTANT MICE
(0.04 MB DOC)

Table S3 MICROARRAY EXPERIMENTAL DESIGN
(0.05 MB DOC)

Table S4 LIST OF GENES DIFFERENTIALLY EXPRESSED (4 FOLD OR GREATER) IN DKO EC BY MICROARRAY ANALYSIS
(0.32 MB DOC)

Table S5 LIST OF GENES DIFFERENTIALLY EXPRESSED IN DKO AORTIC AND LUNG EC BY CONFIRMED BY qPCR ANALYSIS
(0.07 MB DOC)

Figure S1 (A) Schematic illustration of the breeding strategies to obtain *Erk1^−/−^;Erk2^fl/fl^;Tie2-Cre* mutant embryos. Solid triangles represent the loxP sites. (B) Bar graph indicating the number of somites in E9.5 embryos with 3 (grey), 2(white), 1(hatched) and 0 (black) copies of Erk. (C) Graphic panel indicates the ratio of ERK positive to total non-EC, expressed as percent positive non-EC types such as smooth muscle cells, epithelial cells, cardiomyocytes etc. (D) Freshly dissected yolk sac from E9.5 embryos (top) and micrographs of anti-CD31 stained yolk sacs (bottom) from control (left panel) and *Erk1^−/−^;Erk2^fl/fl^;Tie2-Cre* (EC-DKO) mutant (right panel) embryos. Top panel: Bars = 0.25 mm and Bottom panel: Bars = 50µm. (E) Representative paraffin sections from E9.5 control (left, n = 3) and EC-DKO (right, n = 3) embryo sections stained with nuclear fast red. The arrowheads indicate the cellularity of heart. Bars = 20 µm. (F) Head regions from E9.5 embryos analyzed by whole mount staining with anti-CD31 antibody. Controls are in the top row and EC-DKO mutants in the bottom row. Arrowheads highlight examples of blood vessel staining and branching in the controls that are reduced in the EC-DKO embryos.
(3.45 MB TIF)

Figure S2 (A) Typical high speed FACS analysis for cells isolated from *Erk1^−/−^;Erk2^fl/fl^* mice using PE-labeled Di-I-Ac-LDL. Pink curve (M1) shows FACS for the PE-negative population. Purple curve (M2) shows FACS for the PE-positive EC population. The blue curve shows the M2 population that was sorted and collected, and then reanalyzed by FACS. (B) PECAM (green) and DAPI (blue) staining of aortic EC. The third figure in the panel is the merged imaged as indicated. Bars = 20 µm. (C) Anti-ERK1/2 staining on cultured aortic EC of the indicated genotype, without (top panel) and with (bottom panel) Cre. Red-ERK1/2 and Blue-DaPI. Bars = 20 µm. (D) Western blot analysis on lysates from *Erk1^+/−^;Erk2^fl/fl^*-Cre (lane 1), *Erk1^−/−^;Erk2^fl/fl^*-Cre (lane 2) and *Erk1^−/−^;Erk2^fl/fl^*+Cre lung EC for ERK1/2. (E) Gene expression analysis of transcription regulators by q-PCR in aortic EC with and without ERK1/2. (F) Putative target gene expression analysis aortic EC with and without ERK1/2. Note that the expression of these genes was not affected by Erk1/2 status. (G) Gene expression analysis of cell cycle/proliferation, transcription and cell migration/ECM remodeling/angiogenesis regulators downregulated (left panel) and upregulated (right panel) by qPCR in cultured lung EC with and without ERK1/2. ND-not detected.
(25.27 MB TIF)

Figure S3 (A) Graphic panel indicating the ratio of BrDU positive to total non-EC types such as smooth muscle cells, epithelial cells, cardiomyocytes etc., expressed as percent positive non-EC. (B) Anti-BrDU staining on cultured lung EC of the indicated genotype, without (top panel) and with (bottom panel) Cre. Red-BrDU and Blue-DaPI. Bars = 20 µm. The graph shows the quantification of the staining data represented as % BrDU positive EC. (C) Anti-BrDU staining on cultured aortic EC of the indicated genotype, without (top panel, 3 Erk copies) and with (bottom panel,1 Erk copy) Cre. Red-BrDU and Blue-DaPI. Bars = 20 µm. The graph shows the quantification of the staining data represented as % BrDU positive EC. (D) Live/Dead staining of control (top panels) and DKO (bottom panels) aortic EC. GFP-lentivirus infected cells and Red-EthD1/apoptotic cells. Graph at right is quantification of the results. Bars = 40 µm. (E) Senescence associated β-gal staining on control (top) and mutant (bottom) aortic EC before (left panels) and after (right panels) UV induction. Bars = 100 µm. The bar graph indicates the quantification of the results represented as % β-gal positive EC.
(4.52 MB TIF)

Figure S4 (A) Confluent monolayer of control (top panel) and DKO (bottom panel) lung EC were wounded and wound closure was monitored over 24 hrs. Representative results from t = 0 h (left panel) and t = 24 h (right panel) are shown. Graph on the right illustrates the quantification of the number of cells migrating into the wound over the indicated time points. Bars = 40 µm. (B) Confluent monolayer of aortic EC of the indicated genotype, without (top panel, 3 Erk copies) and with (bottom panel,1 Erk copy) Cre were wounded and wound closure was monitored over 24 hrs. Representative results from t = 0 h (left panel) and t = 24 h (right panel) are shown. Graph on the right illustrates the quantification of the number of cells migrating into the wound over the indicated time points. Bars = 40 µm. (C) Migration track assay on control (left) and a mixed population of DKO (right) aortic EC. The GFP expressing cells in the right panel are the mutant EC lacking both Erk1/2 and the non-GFP cells are the uninfected population of control EC. Graphic panel indicates the quantification of the non-fluorescent tracks represented as average area migrated per cell in %.
(1.93 MB TIF)

Figure S5 (A) Immunofluorescence micrographs of actin staining in control (top) and DKO (bottom) lung EC by phalloidin staining. Red-Actin and Green-lentivirus infected EC. Bars = 20 µm. (B) Gene expression analysis of *Paxillin* and *Fak* by q-PCR in aortic EC of the indicated genotype. (C) Western blot analysis on lysates from *Erk1^+/−^;Erk2^fl/fl^*-Cre (lane 1), *Erk1^−/−^;Erk2^fl/fl^*-Cre (lane 2) and *Erk1^−/−^;Erk2^fl/fl^*+Cre lung EC for pS473-AKT. (D) Graphic panels indicate the ratio of Paxillin (left) and FAK (right) positive to total non-EC (such as smooth muscle cells, epithelial cells, cardiomyocytes etc) area respectively in %.
(1.39 MB TIF)

Movie S1 Wound Healing Assay on Erk1/2 Wild type endothelial cells.
(14.07 MB AVI)

Movie S2 Wound Healing Assay on Erk1/2 DKO endothelial cells.
(13.69 MB AVI)
