# Supplementary material for: Erk1 and Erk2 Regulate Endothelial Cell Proliferation and Migration during Mouse Embryonic Angiogenesis
Source: PLoS One. 2009 Dec 14;4(12):e8283. doi: 10.1371/journal.pone.0008283 (PMC2789384; doi:10.1371/journal.pone.0008283)
Supplement: Table S1 — EMBRYONIC LETHALITY IN Erk1−/−; Erk2fl/fl; Tie2Cre DOUBLE MUTANT MICE (0.05 MB DOC) [file pone.0008283.s001.doc]

**SUPPLEMENTARY TABLE 1**. **EMBRYONIC LETHALITY IN** ***Erk1^-/-^; Erk2^fl/fl^; Tie2Cre*DOUBLE MUTANT MICE**

*GENETIC CROSS: Erk1^+/-^; Erk2^fl/+^; Tie2-Cre/+ ♂ X Erk1^-/-^; Erk2^fl/fl^; +/+ ♀*

| **Genotype** | **E9.5** | | **E10.5** | | **P10** | |
| --- | --- | --- | --- | --- | --- | --- |
|  | *Expected* | *Observed* | *Expected* | *Observed* | *Expected* | *Observed* |
| ***Erk1^-/-^;Erk2^fl/fl^;Tie2-Cre*** | 4 | 5 | 4.85 | 4* | 15.125 | 0* |
| ***Erk1^-/-^;Erk2^fl/+^;Tie2-Cre*** | 4 | 1 | 4.85 | 6 | 15.125 | 15 |
| ***Erk1^-/-^;Erk2^fl/fl^*** | 4 | 7 | 4.85 | 4 | 15.125 | 20 |
| ***Erk1^-/-^;Erk2^fl/+^*** | 4 | 1 | 4.85 | 10 | 15.125 | 19 |
| ***Erk1^+/-^;Erk2^fl/fl^;Tie2-Cre*** | 4 | 4 | 4.85 | 6 | 15.125 | 20 |
| ***Erk1^+/-^;Erk2^fl/+^;Tie2-Cre*** | 4 | 5 | 4.85 | 1 | 15.125 | 13 |
| ***Erk1^+/-^;Erk2^fl/fl^*** | 4 | 7 | 4.85 | 6 | 15.125 | 19 |
| ***Erk1^+/-^;Erk2^fl/+^*** | 4 | 2 | 4.85 | 2 | 15.125 | 15 |
| ***Total*** | 32 | | 39 | | 121 | |

* Dead or absent embryos/mice
