# Supplementary material for: Erk1 and Erk2 Regulate Endothelial Cell Proliferation and Migration during Mouse Embryonic Angiogenesis
Source: PLoS One. 2009 Dec 14;4(12):e8283. doi: 10.1371/journal.pone.0008283 (PMC2789384; doi:10.1371/journal.pone.0008283)
Supplement: Table S2 — EMBRYONIC LETHALITY IN Erk1−/−; Erk2fl/fl; Tie2Cre DOUBLE MUTANT MICE (0.04 MB DOC) [file pone.0008283.s002.doc]

**SUPPLEMENTARY TABLE 2**. **EMBRYONIC LETHALITY IN** ***Erk1^-/-^; Erk2^fl/fl^; Tie2Cre*DOUBLE MUTANT MICE**

*GENETIC CROSS: Erk1^-/-^; Erk2^fl/+^; Tie2-Cre/+ ♂ X Erk1^-/-^; Erk2^fl/fl^; +/+ ♀*

|  | **Total** | ***Erk1;Erk2^fl/fl^;Tie2Cre*** | ***Erk1;Erk2^fl/+^;Tie2Cre*** | ***Erk1;Erk2^fl/fl^;+/+*** | ***Erk1;Erk2^fl/+^;+/+*** |
| --- | --- | --- | --- | --- | --- |
| **# Embryos at E9.5** | 136 |  |  |  |  |
| Expected # |  | 34 | 34 | 34 | 34 |
| Actual # |  | 32 | 36 | 31 | 37 |
| **# Embryos at E10.5** | 35 |  |  |  |  |
| Expected # |  | 8.75 | 8.75 | 8.75 | 8.75 |
| Actual # |  | 9* | 7 | 6 | 13 |

***** Dead or absent embryos/mice
