# Supplementary material for: Erk1 and Erk2 Regulate Endothelial Cell Proliferation and Migration during Mouse Embryonic Angiogenesis
Source: PLoS One. 2009 Dec 14;4(12):e8283. doi: 10.1371/journal.pone.0008283 (PMC2789384; doi:10.1371/journal.pone.0008283)
Supplement: Table S3 — MICROARRAY EXPERIMENTAL DESIGN (0.05 MB DOC) [file pone.0008283.s003.doc]

**SUPPLEMENTARY TABLE 3**. **MICROARRAY EXPERIMENTAL DESIGN**

| **TITLE** | | | Expression Data from ERK1/2 null endothelial cells (EC) | | | | |
| --- | --- | --- | --- | --- | --- | --- | --- |
| **STUDY DESIGN** | | | Lentivirus infected cells to generate ERK1/2 WT and ERK1/2 DKO endothelial cells were cultured, RNA was extracted and Affymetrix gene expression arrays were performed. | | | | |
| **SAMPLE INFORMATION:** | | | | | | | |
| ***Organism:*** | *Mus musculus* | | | | | | |
| ***Source Name:*** | Aortic EC isolated from mouse infected with: (a) lentivirus-*egfp-Cre* and (b) lentivirus-*egfp*. | | | | | | |
| ***Strain:*** | C57/BL6 (5 generations) | | | | | | |
| ***Sex:*** | Male | | | | | | |
| ***Age:*** | 4 weeks | | | | | | |
| ***Tissue:*** | Aorta | | | | | | |
| ***Cell Type:*** | Endothelial Cells | | | | | | |
| ***Molecule:*** | Total mRNA | | | | | | |
| ***Label:*** | Biotin | | | | | | |
| ***Sample Name:*** | *Erk1/2DKO-1* | *Erk1/2DKO-2* | | *Erk1/2DKO-3* | *Erk1/2WT-1* | *Erk1/2WT-2* | *Erk1/2WT-3* |
| ***Sample Details:*** | ERK1/2 null EC, biological replicate1 | ERK1/2 null  EC, biological replicate2 | | ERK1/2 null  EC, biological replicate3 | ERK1/2 wild type EC, biological replicate1 | ERK1/2 wild type EC, biological replicate1 | ERK1/2 wild type EC, biological replicate1 |
| ***Genotype:*** | *Erk1^-/-^;Erk2^fl/fl^* + lentiviral-  egfp-Cre | *Erk1^-/^;Erk2^fl/fl^*+  + lentiviral-  egfp-Cre | | *Erk1^-/^;Erk2^fl/fl^* + lentiviral  -egfp-Cre | *Erk1^-/-^;Erk2^fl/fl^* lentiviral-  egfp | *Erk1^-/-^ ;Erk2^fl/fl^* lentiviral-  egfp | *Erk1^-/-^ ;Erk2^fl/fl^* lentiviral-  egfp |
| **ARRAY INFORMATION:** | | | | | | | |
| ***Array Used:*** | Affymetrix Mouse Exon v1.0 st | | | | | | |
| ***Platform:*** | GPL6193 | | | | | | |
|  |  | | | | | | |
| **PROTOCOLS USED:** | | | | | | | |
| ***Growth Protocol*** | EC were isolated from mouse aorta | | | | | | |
| ***Treatment Protocol:*** | EC were grown in complete EC media (DMEM-F12+20% FBS+heparin+ECGS+PS) in a 37º incubator with 5% CO_2_. | | | | | | |
| ***Extraction Protocol:*** | Trizol extraction of total RNA was performed according to the manufacturer's instructions. | | | | | | |
| ***Label Protocol:*** | Biotinylated cRNA was prepared according to the standard Affymetrix protocol from 2 ug total RNA (Expression Analysis Technical Manual, 2007, Affymetrix). | | | | | | |
| ***Hybrization Protocol:*** | Following fragmentation, 15 ug of cRNA was hybridized for 16 hours at 44ºC on Mouse Exon 1.0 ST GeneChips. GeneChips were washed and stained in the Affymetrix Fluidics Station 400. | | | | | | |
| ***Scan protocol:*** | GeneChips were scanned using the Affymetrix GeneChip Scanner 3000 7G | | | | | | |
| ***Data Processing*** | RMA method was applied to the primary data to correct the technical bias and summarize gene expression values over probe-sets | | | | | | |
| ***Value Definition:*** | Log2 expression values | | | | | | |
| **DATA ANALYSIS** | | | | | | | |
| Genes with log2 expression level < 5 for over 80% samples were filtered out. Linear models were performed on the resultant gene set to detect differentially expressed genes between treatment groups. In order to improve the estimates of variability and obtain statistically significant comparisons, variance shrinkage methods were employed for this study (1). The significance levels were adjusted by controlling the mean number of false positives (2). References: (1) Smyth, G.K. (2004), Statistical Applications in Genetics and Molecular Biology 3: Article 3. (2) Gordon, A., Glazko, G., Qiu, X. and Yakovlev, A. (2007), The Annals of Applied Statistics 1:179-190. | | | | | | | |
