# Supplementary material for: Erk1 and Erk2 Regulate Endothelial Cell Proliferation and Migration during Mouse Embryonic Angiogenesis
Source: PLoS One. 2009 Dec 14;4(12):e8283. doi: 10.1371/journal.pone.0008283 (PMC2789384; doi:10.1371/journal.pone.0008283)
Supplement: Table S4 — LIST OF GENES DIFFERENTIALLY EXPRESSED (4 FOLD OR GREATER) IN DKO EC BY MICROARRAY ANALYSIS (0.32 MB DOC) [file pone.0008283.s004.doc]

**SUPPLEMENTARY TABLE 4**. **LIST OF GENES DIFFERENTIALLY EXPRESSED (4 FOLD OR GREATER) IN DKO EC BY MICROARRAY ANALYSIS**

**Cell Cycle/Proliferation Genes**

| **Gene Symbol** | **p-value** | **FoldChange (up_down)** | **Gene Symbol** | **p-value** | **FoldChange (up_down)** |
| --- | --- | --- | --- | --- | --- |
| *Ccnb1* | 0.000371193 | -7.46 | *Birc5* | 0.000009710 | -4.72 |
| *Mki67* | 0.000000225 | -6.78 | *Dbf4* | 0.000084654 | -4.69 |
| *Cenpe* | 0.000285656 | -6.27 | *Dbf4* | 0.000084654 | -4.69 |
| *Cdc25c* | 0.002184239 | -6.06 | *Plk1* | 0.000211792 | -4.68 |
| *Fbxo5* | 0.000047727 | -5.87 | *Ercc6l* | 0.000005327 | -4.59 |
| *Esco2* | 0.000045550 | -5.84 | *Mcm5* | 0.000008852 | -4.55 |
| *Exo1* | .000000424 | -5.64 | *Tpx2* | 0.000043209 | -4.53 |
| *Hells* | 0.000000863 | -5.64 | *Gen1* | 0.000001258 | -4.52 |
| *Ccne2* | 0.000201306 | -5.55 | *Pola1* | 0.000002058 | -4.52 |
| *Kif11* | 0.000010827 | -5.54 | *Rad51* | 0.000006306 | -4.49 |
| *Aspm* | 0.000177687 | -5.46 | *Sgol2* | 0.000019908 | -4.45 |
| *Cdc6* | 0.000015352 | -5.40 | *Rad51ap1* | 0.000001113 | -4.42 |
| *Aurka* | 0.000262518 | -5.39 | *Dtl* | 0.000009369 | -4.40 |
| *Bub1b* | 0.000247982 | -5.39 | *Cdkn3* | 0.000047371 | -4.40 |
| *Bub1* | 0.000247982 | -5.39 | *Mcm7* | 0.000009465 | -4.37 |
| *Spag5* | 0.000013960 | -5.38 | *Cdc2a* | 0.000033566 | -4.35 |
| *Cenph* | 0.000006210 | -5.32 | *Ncaph* | 0.000004387 | -4.34 |
| *Ndc80* | 0.000005075 | -5.24 | *Dscc1* | 0.000027570 | -4.33 |
| *Kif20b* | 0.000011422 | -5.24 | *Smc2* | 0.000001797 | -4.26 |
| *Cep55* | 0.000163175 | -5.19 | *Mcm2* | 0.000024251 | -4.25 |
| *Clspn* | 0.000022695 | -5.13 | *Cdca7l* | 0.000001721 | -4.24 |
| *Ect2* | 0.000030224 | -5.06 | *Cdca7* | 0.000001721 | -4.24 |
| *Tk1* | 0.000015369 | -5.01 | *Sgol1* | 0.000005483 | -4.21 |
| *Anln* | 0.000004921 | -4.98 | *Ccnb2* | 0.007908428 | -4.20 |
| *Nek2* | 0.000416823 | -4.96 | *Mad2l1* | 0.000004535 | -4.19 |
| *Kif20a* | 0.000195293 | -4.96 | *Cit* | 0.000079748 | -4.18 |
| *Ncapg2* | 0.000037209 | -4.94 | *Polq* | 0.000003025 | -4.16 |
| *Top2a* | 0.000015277 | -4.93 | *Mcm6* | 0.000214626 | -4.14 |
| *Prim1* | 0.000162689 | -4.86 | *Ncapd2* | 0.000012300 | -4.11 |
| *Nuf2* | 0.000004859 | -4.86 | *Rad54l* | 0.000000551 | -4.08 |
| *Pole* | 0.000004355 | -4.80 | *Fanca* | 0.000001493 | -4.06 |
| *Ccna2* | 0.000004191 | -4.79 | *Incenp* | 0.000017276 | -4.04 |
| *Dlgap5* | 0.000070956 | -4.77 | *Mcm10* | 0.000004080 | -4.02 |
| *Cdca3* | 0.000049483 | -4.77 | *Gadd45b* | 0.005680641 | 4.20 |
| *Prc1* | 0.000002008 | -4.76 | *Gas6* | 0.000908464 | 4.72 |

**Inflammatory Response genes and Transcriptional Regulation genes**

| **Gene Symbol** | **p-value** | **FoldChange (up_down)** | **Gene Symbol** | **p-value** | **FoldChange (up_down)** |
| --- | --- | --- | --- | --- | --- |
| *C3ar1* | 0.000250852 | -4.93 | *Il1rl1* | 0.002694044 | -8.21 |
| *EG317677* | 0.003187093 | 4.40 | *Pbk* | 0.000004923 | -6.33 |
| *Fas* | 0.007721802 | 4.40 | *Prkg2* | 0.000063874 | -6.11 |
| *Ccl20* | 0.032919289 | 5.12 | *E2f8* | 0.000004120 | -6.09 |
| *Clu* | 0.007914345 | 5.47 | *Ttk* | 0.000001276 | -5.87 |
| *Mx2* | 0.024951482 | 5.58 | *Hist1h2bb* | 0.004544353 | -5.45 |
| *C1rb* | 0.000134277 | 5.74 | *Tcfap2c* | 0.006619475 | -5.23 |
| *Islr* | 0.000088774 | 5.81 | *Uhrf1* | 0.000003928 | -5.09 |
| *Ndrg1* | 0.000002656 | 6.33 | *Asf1b* | 0.000007706 | -5.09 |
| *C4b* | 0.000993859 | 7.29 | *Wdhd1* | 0.000002148 | -4.78 |
| *Gbp2* | 0.013523006 | 8.16 | *Melk* | 0.000006824 | -4.77 |
| *Gbp3* | 0.040144040 | 8.35 | *Chaf1b* | 0.000045299 | -4.75 |
| *C1s* | 0.000082562 | 9.35 | *Hist4h4* | 0.001221093 | -4.72 |
| *Cd200* | 0.013845391 | 10.06 | *Suv39h2* | 0.000021914 | -4.70 |
| *C3* | 0.001702987 | 11.46 | *Cenpa* | 0.000618729 | -4.68 |
| *Ly6a* | 0.000655901 | 12.02 | *Ezh2* | 0.000312822 | -4.67 |
| *Ifit1* | 0.018225003 | 15.91 | *Atad2* | 0.000006651 | -4.64 |
| *Cfb* | 0.010537558 | 17.67 | *Brca1* | 0.000001157 | -4.61 |
| *Vnn1* | 0.000547237 | 18.00 | *Mcm3* | 0.000002285 | -4.57 |
| *Hp* | 0.001713321 | 19.33 | *Plk4* | 0.000006488 | -4.54 |
| *Lbp* | 0.000138718 | 25.68 | *Slbp* | 0.001788317 | -4.49 |
| *Saa3* | 0.002715363 | 34.87 | *Foxm1* | 0.000002855 | -4.41 |
|  |  |  | *Hist1h1b* | 0.000707753 | -4.24 |
|  |  |  | *Hist1h1a* | 0.002428483 | -4.22 |
|  |  |  | *Aurkb* | 0.000014554 | -4.14 |
|  |  |  | *E2f7* | 0.000046823 | -4.13 |
|  |  |  | *Mastl* | 0.000017316 | -4.06 |
|  |  |  | *Brip1* | 0.000092739 | -4.03 |
|  |  |  | *Cenpk* | 0.000031128 | -4.01 |
|  |  |  | *Rora* | 0.002387727 | 4.08 |
|  |  |  | *Epha3* | 0.000229718 | 4.24 |
|  |  |  | *Stat2* | 0.003946342 | 4.34 |
|  |  |  | *Atf3* | 0.000298317 | 4.52 |
|  |  |  | *Afap1l2* | 0.001057908 | 4.82 |
|  |  |  | *Pdgfd* | 0.034387219 | 5.04 |
|  |  |  | *Hist1h2bc* | 0.000075192 | 5.47 |
|  |  |  | *Lmcd1* | 0.005133128 | 6.42 |

**Genes regulating Cell migration/ECM remodeling/Angiogenesis, Metabolism and Other Processes**

| **Gene Symbol** | **p-value** | **FoldChange (up_down)** | **Gene Symbol** | **p-value** | **FoldChange (up_down)** |
| --- | --- | --- | --- | --- | --- |
| *Mmp1a* | 0.030998600 | -12.71 | *Lipg* | 0.001682340 | -6.17 |
| *Klhl1* | 0.003367515 | -6.26 | *Pla2g7* | 0.001433961 | -5.61 |
| *Racgap1* | 0.000032250 | -4.53 | *Dhfr* | 0.000026273 | -4.53 |
| *Iqgap3* | 0.000077139 | -4.34 | *Enpp5* | 0.002437951 | 4.12 |
| *Arhgap11a* | 0.000002686 | -4.25 | *Gda* | 0.001865580 | 4.16 |
| *Ckap2l* | 0.000036066 | -4.14 | *Aox3* | 0.001420144 | 4.30 |
| *Tacc3* | 0.000020712 | -4.11 | *Gdpd2* | 0.002409436 | 4.49 |
| *Mmp3* | 0.005211595 | -4.05 | *Cbr2* | 0.006265492 | 5.11 |
| *Diap3* | 0.000014603 | -4.04 | *Sod3* | 0.000352957 | 5.38 |
| *Sprr1a* | 0.002154623 | -4.04 | *Chi3l1* | 0.041065338 | 5.53 |
| *Fgl2* | 0.011098546 | 4.06 | *Mgll* | 0.000036687 | 5.56 |
| *Gadd45g* | 0.003047961 | 4.20 | *Smpdl3a* | 0.000081101 | 5.78 |
| *Reck* | 0.001666196 | 4.39 | *Tgm2* | 0.000104284 | 6.09 |
| *Col3a1* | 0.000131814 | 4.50 | *Loxl2* | 0.005854772 | 6.53 |
| *Epas1* | 0.006335975 | 4.54 | *Fabp3* | 0.000808300 | 6.54 |
| *Nid2* | 0.000002987 | 4.59 | *Podn* | 0.002459442 | 6.62 |
| *Itga8* | 0.030258569 | 4.79 | *Maob* | 0.026436071 | 6.68 |
| *Sepp1* | 0.000493793 | 5.21 | *Serpinb9b* | 0.009149751 | 6.82 |
| *Notch3* | 0.000239452 | 5.30 | *Serpinb9* | 0.000156121 | 6.97 |
| *Angpt2* | 0.001999152 | 5.55 | *Akr1c14* | 0.000043692 | 7.39 |
| *Bmp4* | 0.018615637 | 5.63 | *Fmo2* | 0.000456427 | 9.60 |
| *Fgf18* | 0.003166912 | 5.71 | *Serpina3n* | 0.000012789 | 10.87 |
| *Prkg1* | 0.000066613 | 5.83 | *Fmo1* | 0.000234910 | 10.94 |
| *Lims2* | 0.000339876 | 5.97 | *Steap4* | 0.007767767 | 14.11 |
| *Adamts1* | 0.000111130 | 6.31 | *Cdo1* | 0.000322773 | 16.16 |
| *Ccdc80* | 0.000089031 | 6.40 | *Stil* | 0.000086514 | -4.67 |
| *Scel* | 0.002830029 | 6.64 | *Rlbp1l1* | 0.007215465 | -4.55 |
| *Prss23* | 0.000296197 | 6.69 | *Unc13c* | 0.014724377 | -4.36 |
| *Mtss1* | 0.003445681 | 7.17 | *Kif22* | 0.000006795 | -4.07 |
| *Svep1* | 0.004170478 | 7.62 | *Lgals9* | 0.005585652 | 4.02 |
| *Jag1* | 0.001279392 | 7.95 | *Dusp8* | 0.001324563 | 4.08 |
| *Itgbl1* | 0.001191643 | 8.06 | *Kcnab1* | 0.006336060 | 4.17 |
| *Cldn1* | 0.000119606 | 8.21 | *Casp4* | 0.010153453 | 4.17 |
| *Figf* | 0.000000959 | 8.33 | *Pltp* | 0.000275789 | 4.26 |
| *Serpinb6b* | 0.001412292 | 8.39 | *Cp* | 0.025220114 | 4.32 |
| *Tgfb2* | 0.001043744 | 9.73 | *Slc7a2* | 0.027929837 | 4.34 |
| *Serpinb1a* | 0.000048744 | 10.19 | *Rcan2* | 0.000127458 | 4.34 |
| *Serpina3f* | 0.003985607 | 10.38 | *Myom1* | 0.019185403 | 4.34 |
| *Sfrp2* | 0.001549563 | 11.00 | *Cstad* | 0.000528055 | 4.38 |
| *Wisp2* | 0.000149061 | 11.04 | *Anxa8* | 0.001892803 | 4.52 |
| *Mme* | 0.000037652 | 12.41 | *Tcn2* | 0.000723969 | 4.90 |
| *Eln* | 0.000113166 | 13.32 | *Palm2* | 0.000018698 | 5.02 |
| *Ogn* | 0.008672809 | 14.67 | *Dusp1* | 0.001384066 | 5.34 |
| *Adamts5* | 0.000046877 | 15.75 | *Sema3d* | 0.006267976 | 6.49 |
| *Serping1* | 0.000023693 | 17.69 | *Mrgprf* | 0.002222946 | 7.09 |
| *Fbln5* | 0.000002188 | 19.19 | *Atp2b4* | 0.002226910 | 7.34 |
| *Prelp* | 0.000022232 | 42.49 | *Nkain4* | 0.000874794 | 7.73 |
|  |  |  | *Dpt* | 0.011325436 | 10.28 |
|  |  |  | *Rasl11b* | 0.000282381 | 11.68 |
|  |  |  | *Fhl1* | 0.000892618 | 12.27 |
|  |  |  | *Clca2* | 0.012849617 | 12.38 |
|  |  |  | *Nov* | 0.000131551 | 14.64 |

**Unannotated genes**

| **Gene Symbol** | **p-value** | **FoldChange (up_down)** | **Gene Symbol** | **p-value** | **FoldChange (up_down)** |
| --- | --- | --- | --- | --- | --- |
| *Kif2c* | 0.000428709 | -6.05 | *9430028L06Rik* | 0.001990935 | -4.22 |
| *2810417H13Rik* | 0.000028234 | -6.02 | *Depdc1b* | 0.000015817 | -4.16 |
| *Kif4* | 0.000011449 | -5.55 | *AI504432* | 0.001683585 | -4.01 |
| *Prr11* | 0.000145050 | -5.50 | *Pi15* | 0.010437760 | 4.40 |
| *Cenpi* | 0.000003977 | -5.37 | *S3-12* | 0.011287393 | 4.50 |
| *Casc5* | 0.000002212 | -5.32 | *I830012O16Rik* | 0.023762644 | 4.59 |
| *Shcbp1* | 0.000002255 | -5.23 | *Tmem119* | 0.000240772 | 4.82 |
| *Slc14a1* | 0.000060448 | -5.19 | *Calml4* | 0.036802601 | 4.87 |
| *C79407* | 0.000024689 | -5.14 | *EG240327* | 0.038116696 | 4.92 |
| *Kif15* | 0.000009365 | -5.13 | *Tmem184a* | 0.000072337 | 5.01 |
| *C330027C09Rik* | 0.000477222 | -5.09 | *Niban* | 0.001996854 | 5.14 |
| *D17H6S56E-5* | 0.000119825 | -4.91 | *Mustn1* | 0.001171748 | 5.19 |
| *Hmmr* | 0.000237628 | -4.89 | *Mamdc2* | 0.000435644 | 5.20 |
| *3000004C01Rik* | 0.000005953 | -4.79 | *9230105E10Rik* | 0.000435644 | 5.20 |
| *4930547N16Rik* | 0.000021306 | -4.74 | *Igtp* | 0.005657779 | 5.42 |
| *F630043A04Rik* | 0.000009733 | -4.73 | *1110018M03Rik* | 0.004350272 | 5.47 |
| *Lrrtm2* | 0.000748240 | -4.62 | *Tmem140* | 0.000039630 | 5.77 |
| *Ubash3b* | 0.005480075 | -4.54 | *1200015N20Rik* | 0.004879350 | 6.59 |
| *Nptx1* | 0.034174012 | -4.52 | *ORF63* | 0.000071771 | 7.11 |
| *Areg* | 0.023128350 | -4.48 | *Mfap5* | 0.005198583 | 7.44 |
| *5730590G19Rik* | 0.000010408 | -4.45 | *Sorbs2* | 0.000827362 | 7.53 |
| *Mdga2* | 0.009026269 | -4.34 | *Pkhd1l1* | 0.001123297 | 7.84 |
| *4632417K18Rik* | 0.000002517 | -4.30 | *Susd2* | 0.000167859 | 7.93 |
| *Cenpn* | 0.000011034 | -4.30 | *Mettl7a1* | 0.000182603 | 8.22 |
| *Fignl1* | 0.000002672 | -4.29 | *Tmtc1* | 0.000061583 | 9.28 |
| *Cenpq* | 0.000136105 | -4.27 | *Prg4* | 0.014317344 | 13.48 |
|  |  |  | *9930013L23Rik* | 0.003767281 | 17.45 |
