# Supplementary material for: Erk1 and Erk2 Regulate Endothelial Cell Proliferation and Migration during Mouse Embryonic Angiogenesis
Source: PLoS One. 2009 Dec 14;4(12):e8283. doi: 10.1371/journal.pone.0008283 (PMC2789384; doi:10.1371/journal.pone.0008283)
Supplement: Table S5 — LIST OF GENES DIFFERENTIALLY EXPRESSED IN DKO AORTIC AND LUNG EC BY CONFIRMED BY qPCR ANALYSIS (0.07 MB DOC) [file pone.0008283.s005.doc]

**SUPPLEMENTARY TABLE 5**. **LIST OF GENES DIFFERENTIALLY EXPRESSED IN DKO AORTIC AND LUNG EC BY CONFIRMED BY qPCR ANALYSIS**

| **Gene Symbol** | **Differential expression in aortic EC** | **Differential expression in lung EC** |
| --- | --- | --- |
| *Anln* | **+** | **+** |
| *Ccna* | **+** |  |
| *Ccnb1* | **+** |  |
| *Ccne* | **+** | **+** |
| *Cdk1* | **+** |  |
| *Cdc6* | **+** | **+** |
| *Exo1* | **+** |  |
| *Hells* | **+** | **+** |
| *Pola1* | **+** |  |
| *Rad54l* | **+** |  |
| *Aurka* | **+** | **+** |
| *Mcm2* | **+** |  |
| *Gadd54β* | **+** | **+** |
| *Gas6* | **+** | **+** |
| *Ckap2l* | **+** | **+** |
| *Diap3* | **+** | **+** |
| *Klhl1* | **+** | **ND** |
| *Mmp1a* | **+** |  |
| *Sfrp2* | **+** | **+** |
| *TGFβ2* | **+** |  |
| *Wisp2* | **+** | **+** |
| *Reck* | **+** | **+** |
| *Prelp* | **+** | **+** |
| *Adamts1* | **+** | **+** |
| *Adamts5* | **+** | **+** |
| *Col3a1* | **+** |  |
| *Serpinb6b* | **+** |  |
| *Serpinb1a* | **+** |  |
| *E2F7* | **+** | **+** |
| *E2F8* | **+** | **+** |
| *Suv39h2* | **+** |  |
| *Stat2* | **+** |  |
| *Ezh2* | **+** |  |
| *Atf3* | **+** | **ND** |
| *Lmcd1* | **+** | **+** |

| **Gene Symbol** | **Differential expression in aortic EC** | **Differential expression in lung EC** |
| --- | --- | --- |
| *MMP11* | **+** |  |
| *Sparc* | **+** | **+** |
| *Thbs1* | **+** | **+** |
| *Thbs2* | **+** | **+** |
| *MMP14* | **+** | **+** |
| *Cdh13* | **+** | **+** |
| *MMP9* | **+** | **+** |
| *uPA* | **+** | **+** |
| *Plaur* | **+** | **+** |
| *Cdh5* | **+** |  |
| *MMP10* | **+** | **+** |
| *Itgb1* | **+** | **+** |
| *Itgb3* | **+** | **+** |

ND – not detected
