# Supplementary figures and images for: Erk1 and Erk2 Regulate Endothelial Cell Proliferation and Migration during Mouse Embryonic Angiogenesis
Source: PLoS One. 2009 Dec 14;4(12):e8283. doi: 10.1371/journal.pone.0008283 (PMC2789384; doi:10.1371/journal.pone.0008283)

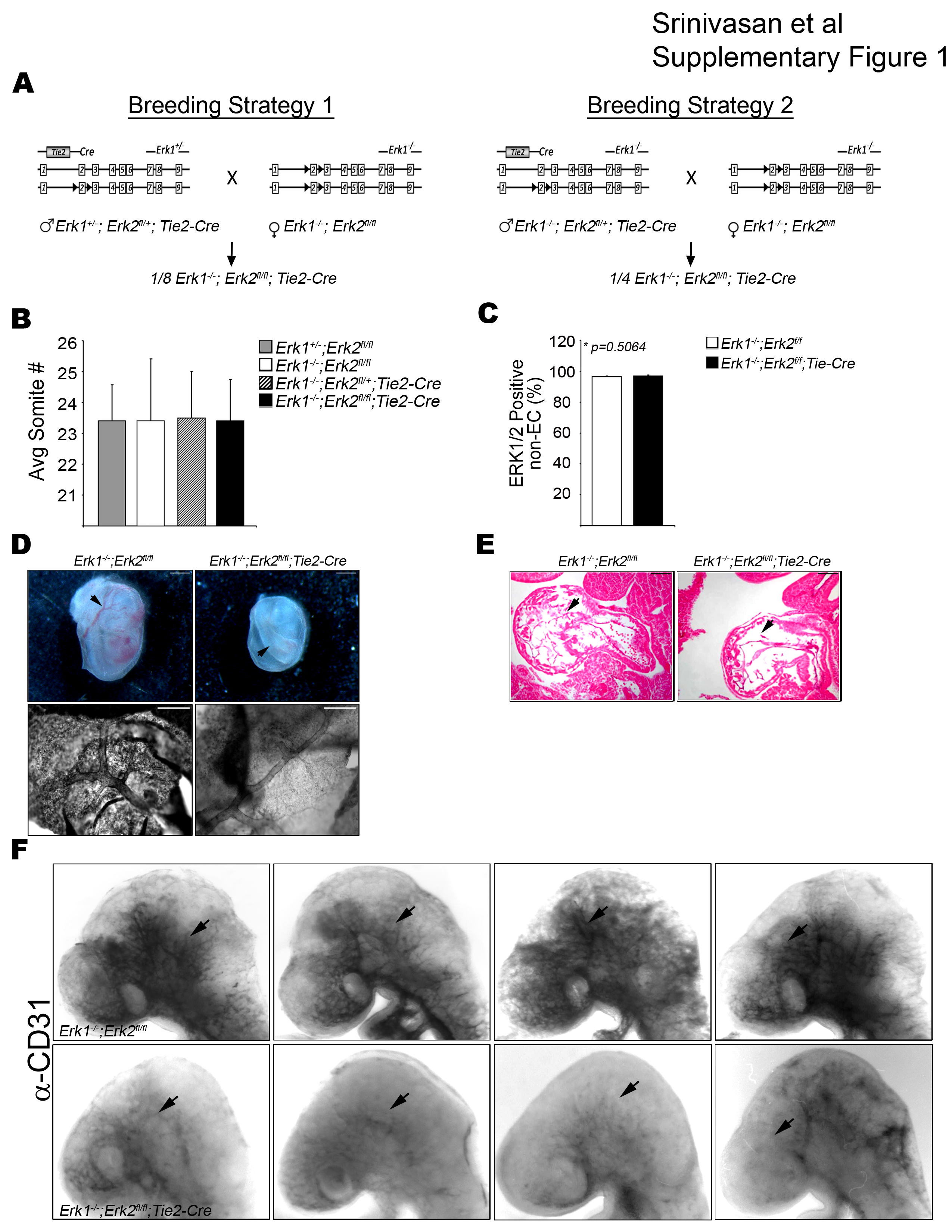

Supplement: Figure S1 — (A) Schematic illustration of the breeding strategies to obtain Erk1−/−;Erk2fl/fl;Tie2-Cre mutant embryos. Solid triangles represent the loxP sites. (B) Bar graph indicating the number of somites in E9.5 embryos with 3 (grey), 2(white), 1(hatched) and 0 (black) copies of Erk. (C) Graphic panel indicates the ratio of ERK positive to total non-EC, expressed as percent positive non-EC types such as smooth muscle cells, epithelial cells, cardiomyocytes etc. (D) Freshly dissected yolk sac from E9.5 embryos (top) and micrographs of anti-CD31 stained yolk sacs (bottom) from control (left panel) and Erk1−/−;Erk2fl/fl;Tie2-Cre (EC-DKO) mutant (right panel) embryos. Top panel: Bars = 0.25 mm and Bottom panel: Bars = 50µm. (E) Representative paraffin sections from E9.5 control (left, n = 3) and EC-DKO (right, n = 3) embryo sections stained with nuclear fast red. The arrowheads indicate the cellularity of heart. Bars = 20 µm. (F) Head regions from E9.5 embryos analyzed by whole mount staining with anti-CD31 antibody. Controls are in the top row and EC-DKO mutants in the bottom row. Arrowheads highlight examples of blood vessel staining and branching in the controls that are reduced in the EC-DKO embryos. (3.45 MB TIF) [file pone.0008283.s006.tif]

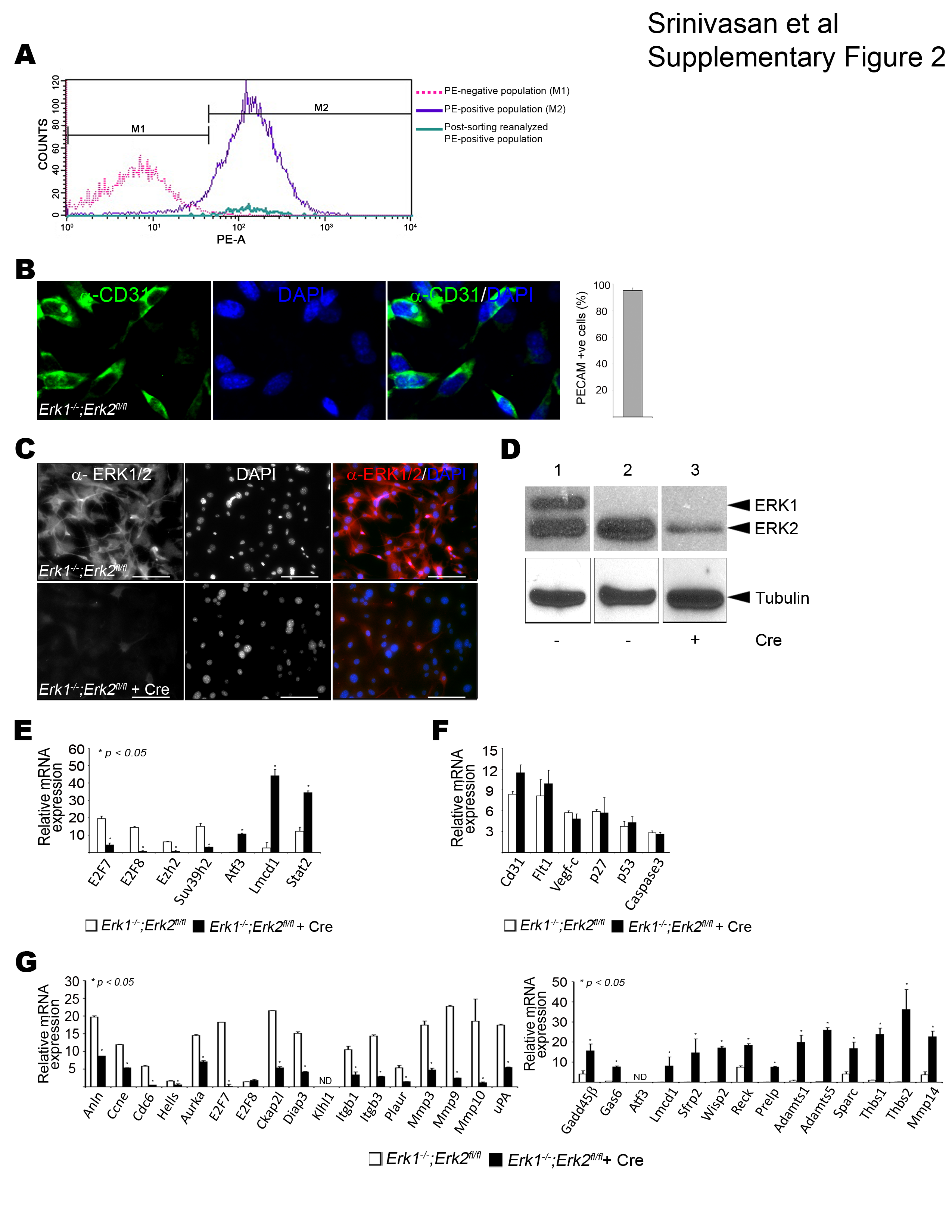

Supplement: Figure S2 — (A) Typical high speed FACS analysis for cells isolated from Erk1−/−;Erk2fl/fl mice using PE-labeled Di-I-Ac-LDL. Pink curve (M1) shows FACS for the PE-negative population. Purple curve (M2) shows FACS for the PE-positive EC population. The blue curve shows the M2 population that was sorted and collected, and then reanalyzed by FACS. (B) PECAM (green) and DAPI (blue) staining of aortic EC. The third figure in the panel is the merged imaged as indicated. Bars = 20 µm. (C) Anti-ERK1/2 staining on cultured aortic EC of the indicated genotype, without (top panel) and with (bottom panel) Cre. Red-ERK1/2 and Blue-DaPI. Bars = 20 µm. (D) Western blot analysis on lysates from Erk1+/−;Erk2fl/fl-Cre (lane 1), Erk1−/−;Erk2fl/fl-Cre (lane 2) and Erk1−/−;Erk2fl/fl+Cre lung EC for ERK1/2. (E) Gene expression analysis of transcription regulators by q-PCR in aortic EC with and without ERK1/2. (F) Putative target gene expression analysis aortic EC with and without ERK1/2. Note that the expression of these genes was not affected by Erk1/2 status. (G) Gene expression analysis of cell cycle/proliferation, transcription and cell migration/ECM remodeling/angiogenesis regulators downregulated (left panel) and upregulated (right panel) by qPCR in cultured lung EC with and without ERK1/2. ND-not detected. (25.27 MB TIF) [file pone.0008283.s007.tif]

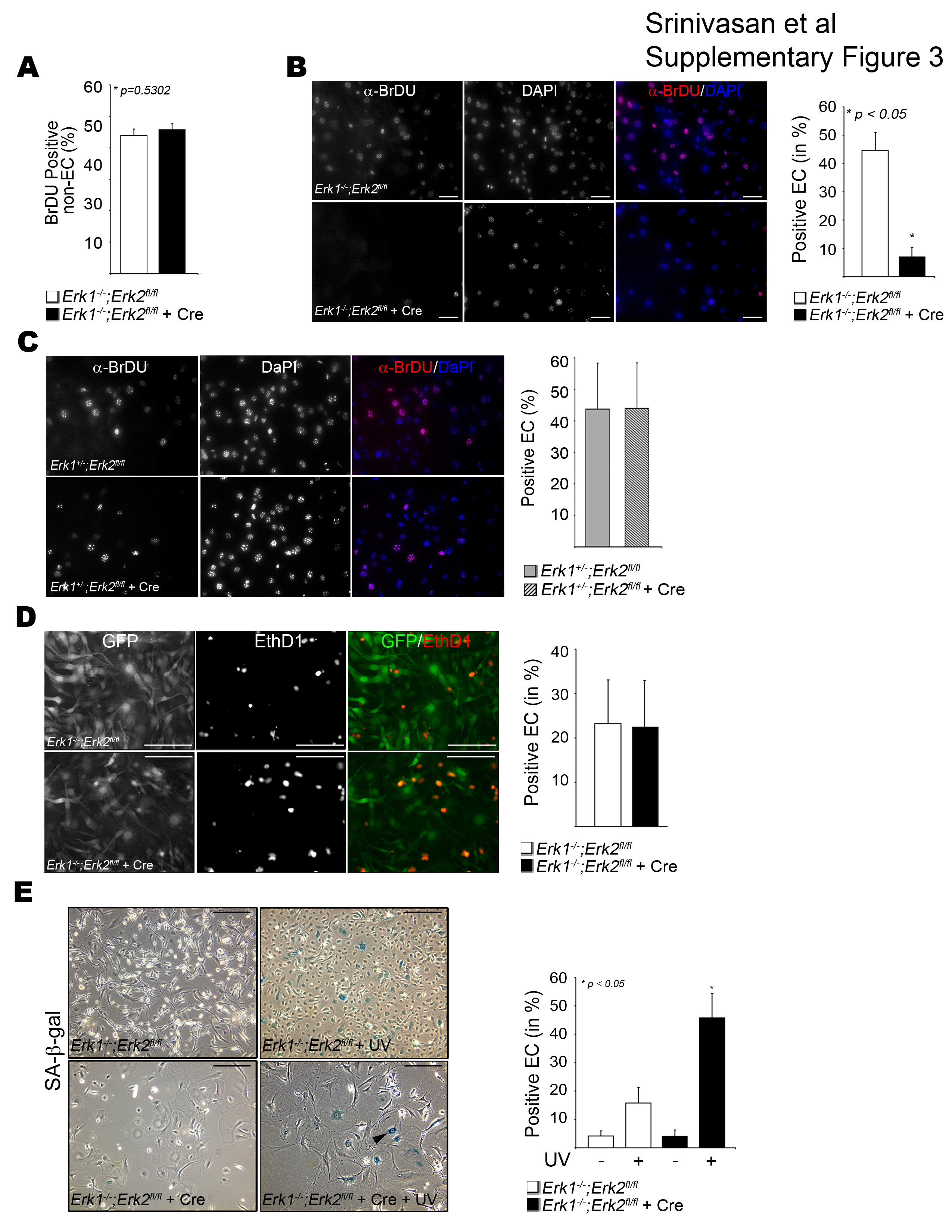

Supplement: Figure S3 — (A) Graphic panel indicating the ratio of BrDU positive to total non-EC types such as smooth muscle cells, epithelial cells, cardiomyocytes etc., expressed as percent positive non-EC. (B) Anti-BrDU staining on cultured lung EC of the indicated genotype, without (top panel) and with (bottom panel) Cre. Red-BrDU and Blue-DaPI. Bars = 20 µm. The graph shows the quantification of the staining data represented as % BrDU positive EC. (C) Anti-BrDU staining on cultured aortic EC of the indicated genotype, without (top panel, 3 Erk copies) and with (bottom panel,1 Erk copy) Cre. Red-BrDU and Blue-DaPI. Bars = 20 µm. The graph shows the quantification of the staining data represented as % BrDU positive EC. (D) Live/Dead staining of control (top panels) and DKO (bottom panels) aortic EC. GFP-lentivirus infected cells and Red-EthD1/apoptotic cells. Graph at right is quantification of the results. Bars = 40 µm. (E) Senescence associated β-gal staining on control (top) and mutant (bottom) aortic EC before (left panels) and after (right panels) UV induction. Bars = 100 µm. The bar graph indicates the quantification of the results represented as % β-gal positive EC. (4.52 MB TIF) [file pone.0008283.s008.tif]

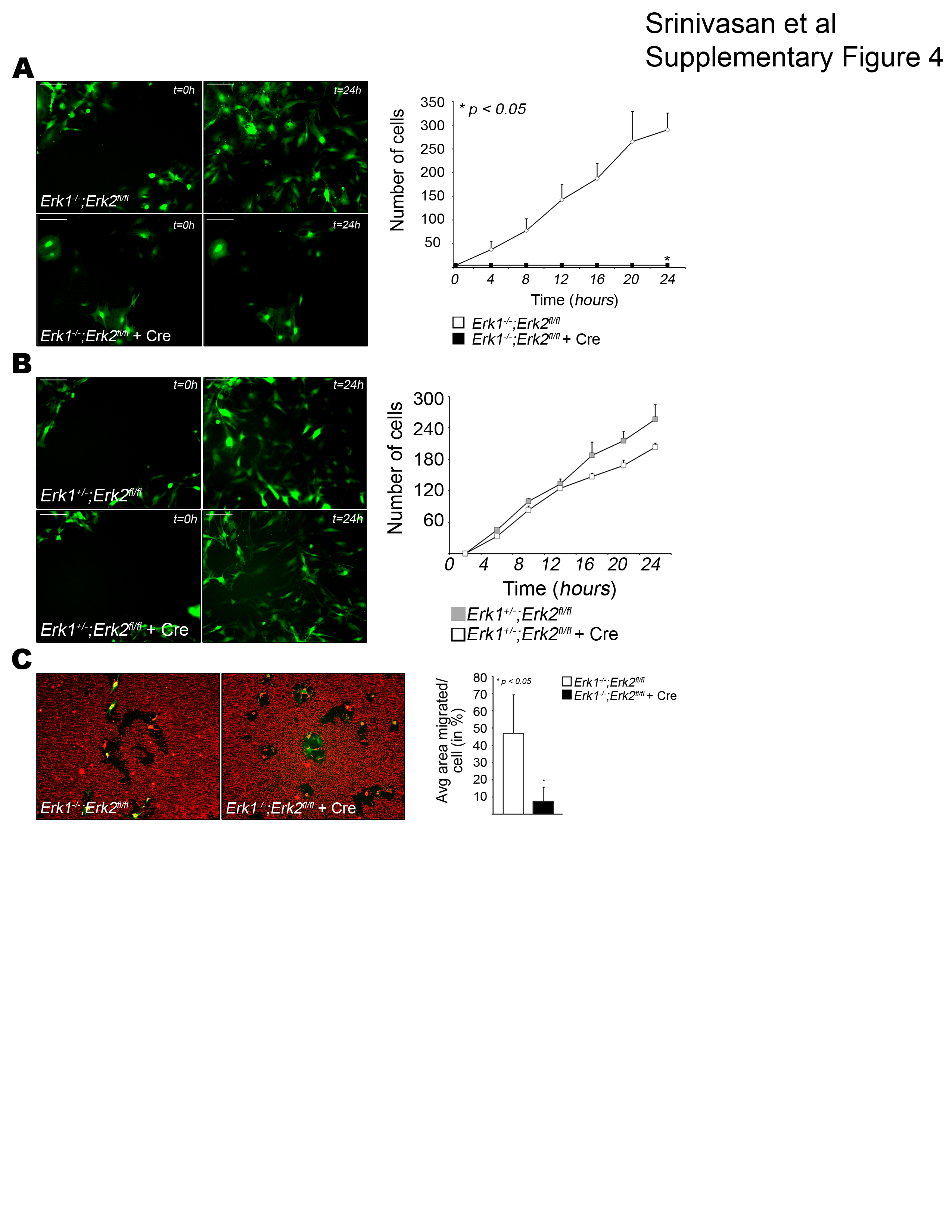

Supplement: Figure S4 — (A) Confluent monolayer of control (top panel) and DKO (bottom panel) lung EC were wounded and wound closure was monitored over 24 hrs. Representative results from t = 0 h (left panel) and t = 24 h (right panel) are shown. Graph on the right illustrates the quantification of the number of cells migrating into the wound over the indicated time points. Bars = 40 µm. (B) Confluent monolayer of aortic EC of the indicated genotype, without (top panel, 3 Erk copies) and with (bottom panel,1 Erk copy) Cre were wounded and wound closure was monitored over 24 hrs. Representative results from t = 0 h (left panel) and t = 24 h (right panel) are shown. Graph on the right illustrates the quantification of the number of cells migrating into the wound over the indicated time points. Bars = 40 µm. (C) Migration track assay on control (left) and a mixed population of DKO (right) aortic EC. The GFP expressing cells in the right panel are the mutant EC lacking both Erk1/2 and the non-GFP cells are the uninfected population of control EC. Graphic panel indicates the quantification of the non-fluorescent tracks represented as average area migrated per cell in %. (1.93 MB TIF) [file pone.0008283.s009.tif]

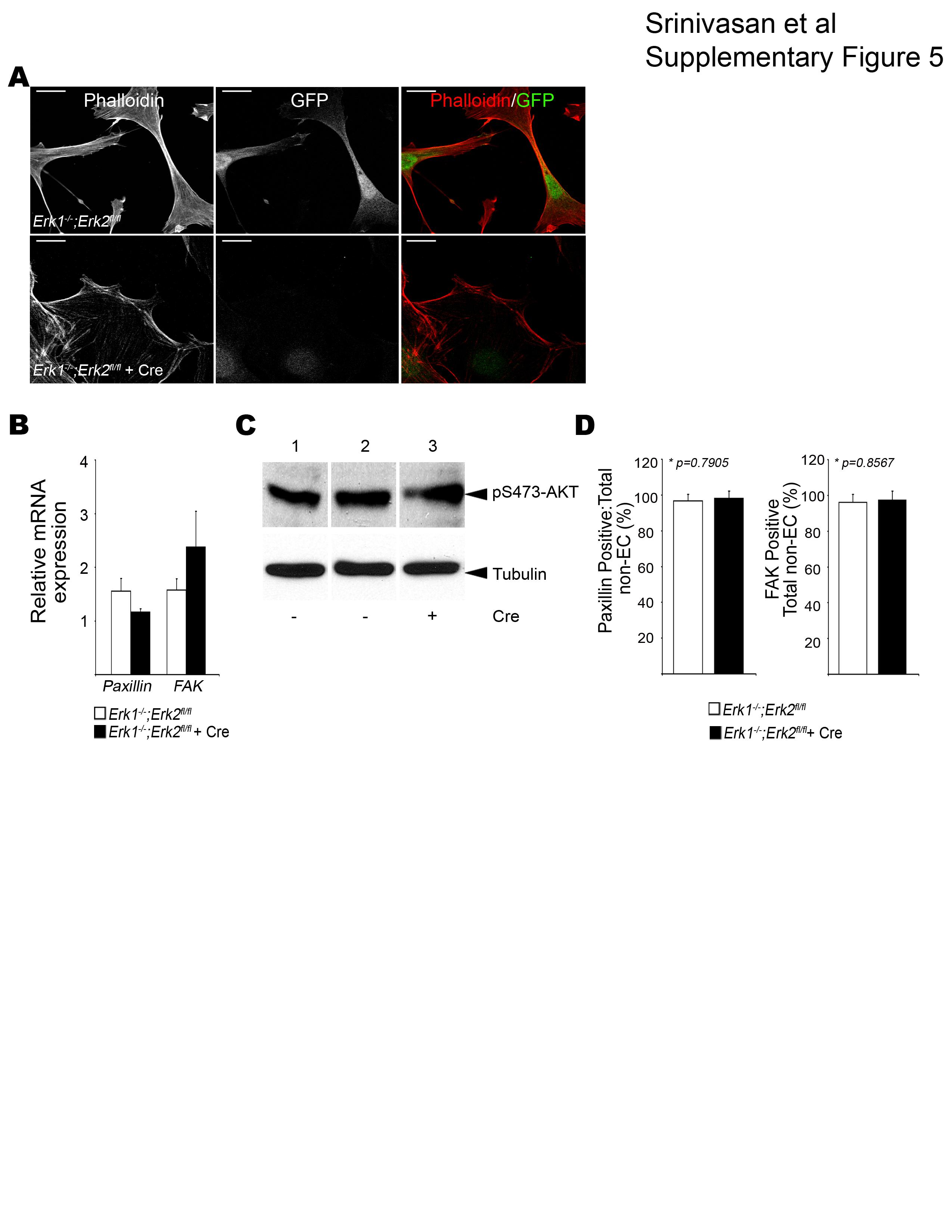

Supplement: Figure S5 — (A) Immunofluorescence micrographs of actin staining in control (top) and DKO (bottom) lung EC by phalloidin staining. Red-Actin and Green-lentivirus infected EC. Bars = 20 µm. (B) Gene expression analysis of Paxillin and Fak by q-PCR in aortic EC of the indicated genotype. (C) Western blot analysis on lysates from Erk1+/−;Erk2fl/fl-Cre (lane 1), Erk1−/−;Erk2fl/fl-Cre (lane 2) and Erk1−/−;Erk2fl/fl+Cre lung EC for pS473-AKT. (D) Graphic panels indicate the ratio of Paxillin (left) and FAK (right) positive to total non-EC (such as smooth muscle cells, epithelial cells, cardiomyocytes etc) area respectively in %. (1.39 MB TIF) [file pone.0008283.s010.tif]
